# *PKD1* and *PKD2* mRNA cis-inhibition drives polycystic kidney disease progression

**DOI:** 10.1038/s41467-022-32543-2

**Published:** 2022-08-15

**Authors:** Ronak Lakhia, Harini Ramalingam, Chun-Mien Chang, Patricia Cobo-Stark, Laurence Biggers, Andrea Flaten, Jesus Alvarez, Tania Valencia, Darren P. Wallace, Edmund C. Lee, Vishal Patel

**Affiliations:** 1 Department of Internal Medicine, Nephrology, UT Southwestern Medical Center, Dallas, TX 75390 USA; 2 Regulus Therapeutics Inc., San Diego, CA 92121 USA; 3 Department of Internal Medicine and the Jared Grantham Kidney Institute, University of Kansas Medical Center, Kansas City, KS USA

**Keywords:** Polycystic kidney disease, Gene regulation

## Abstract

Autosomal dominant polycystic kidney disease (ADPKD), among the most common human genetic conditions and a frequent etiology of kidney failure, is primarily caused by heterozygous *PKD1* mutations. Kidney cyst formation occurs when *PKD1* dosage falls below a critical threshold. However, no framework exists to harness the remaining allele or reverse *PKD1* decline. Here, we show that mRNAs produced by the noninactivated *PKD1* allele are repressed via their 3′-UTR miR-17 binding element. Eliminating this motif (*Pkd1*^∆17^) improves mRNA stability, raises Polycystin-1 levels, and alleviates cyst growth in cellular, ex vivo, and mouse PKD models. Remarkably, *Pkd2* is also inhibited via its 3′-UTR miR-17 motif, and *Pkd2*^∆17^-induced Polycystin-2 derepression retards cyst growth in *Pkd1*-mutant models. Moreover, acutely blocking *Pkd1/2* cis-inhibition, including after cyst onset, attenuates murine PKD. Finally, modeling *PKD1*^∆17^ or *PKD2*^∆17^ alleles in patient-derived primary ADPKD cultures leads to smaller cysts, reduced proliferation, lower pCreb1 expression, and improved mitochondrial membrane potential. Thus, evading 3′-UTR cis-interference and enhancing *PKD1/2* mRNA translation is a potentially mutation-agnostic ADPKD-arresting approach.

## Abstract (web summary)

ADPKD, a common aetiology of kidney failure, is caused by heterozygous *PKD1* or *PKD2* mutations. Here the authors show that preventing 3′-UTR cis-inhibition of mRNAs produced by the non-inactivated *PKD1/2* alleles ameliorates preclinical ADPKD.

## Introduction

An estimated 12.5 million people worldwide suffer from autosomal dominant polycystic kidney disease (ADPKD), making it among the most common monogenetic conditions known to humankind. A clinical hallmark of ADPKD is the relentless growth of innumerable fluid-filled cysts in the kidneys, which replace the normal parenchyma and, over decades, cause massive bilateral kidney enlargement and renal failure^1^. ADPKD occurs because of heterozygous, loss-of-function mutations in *PKD1* (~78% of cases) or *PKD2* (~15% of cases). The classical hypothesis for cyst initiation is that in addition to a germline inactivating mutation in one allele of the PKD gene, there is somatic inactivation (referred to as the second hit) in the other allele, causing a complete loss of polycystin expression in the cell. However, in recent years, several lines of evidence support the gene dose threshold as a mechanism involved in cystogenesis^2,3^. This hypothesis posits that complete *PKD1* loss is not necessary, but rather cystogenesis ensues if the functional *PKD1* dosage falls below a critical threshold. Supporting the gene dosage model, inactivating second hit mutation is not a universal feature, especially in smaller ADPKD cysts^4–7^. Importantly, many individuals with ADPKD continue to have residual PC1 expression because they carry missense (rather than inactivating) germline *PKD1* mutations^8–10^. As proof of principle, lowering the *Pkd1* dose is sufficient to produce PKD in mice, pigs, and monkeys^4,11–16^. Thus, if reduced dosage causes ADPKD, increasing the expression of the normal *PKD1* allele could arrest the disorder. However, despite this transformative potential, the factors governing *PKD1* dosage in ADPKD are mostly unknown, and currently, there are no mechanisms to activate the normal *PKD1* allele.

The 3′-untranslated region (3′-UTR), the mRNA portion that lies immediately downstream of the translation termination codon, protects the mRNA from degradation and facilitates translation through its poly(A) tail^17^. Paradoxically, the 3′-UTR, via interaction with microRNAs (miRNAs), can also mediate mRNA translation repression or deadenylation^18–21^. Most mRNA 3′-UTRs harbor evolutionarily conserved miRNA-binding elements (MBEs), implying that cis-inhibition of translation is a pervasive mode of gene output regulation^22^. However, this intriguing aspect of the 3′-UTR function is poorly delineated. The prediction is that individual MBEs have a minor impact on host mRNA function, considering that miRNAs mostly act as rheostats and modestly repress mRNA targets. Counter to this prevailing logic, we reasoned that under certain circumstances, such as when gene dosage is already reduced due to haploinsufficiency, MBE-mediated cis-inhibition of the remaining allele could have a disease-modifying effect by governing the final protein output.

Our goal was to determine whether monoallelic *PKD1* derepression is possible and how it influences preclinical ADPKD progression. *PKD1* contains a miR-17 binding motif in its 3′-UTR, and miR-17 expression and activity are higher in ADPKD models^4,23–26^. Therefore, we tested the idea that *PKD1* mRNA is cis-inhibited by its 3′-UTR miR-17 motif, and blocking this inhibition reverses *PKD1* decline. We use CRISPR/Cas9 editing to delete the miR-17 motif from the *PKD1* gene in monoallelic ADPKD models. We find that eliminating the miR-17 motif is sufficient to improve *Pkd1* mRNA stability, raise Polycystin-1 (PC1) expression, and ameliorate cyst growth in cellular, ex vivo, and mouse PKD models. The other ADPKD gene, *PKD2*, also contains a 3′-UTR miR-17 binding motif; remarkably, deleting this miR-17 motif increases Polycystin-2 (PC2) levels and attenuates cyst growth in *Pkd1*-mutant models. Furthermore, acute pharmaceutical blockade of *Pkd1/2* cis-inhibition prevents cyst onset and stabilizes established PKD in mice. Finally, we demonstrate that *PKD1* or *PKD2* derepression reverses cyst-pathogenic events in primary kidney cyst epithelia derived from individuals with ADPKD.

## Results

### *Pkd1* is cis-repressed via its 3′-UTR miR-17 binding motif

**Fig. 1:**
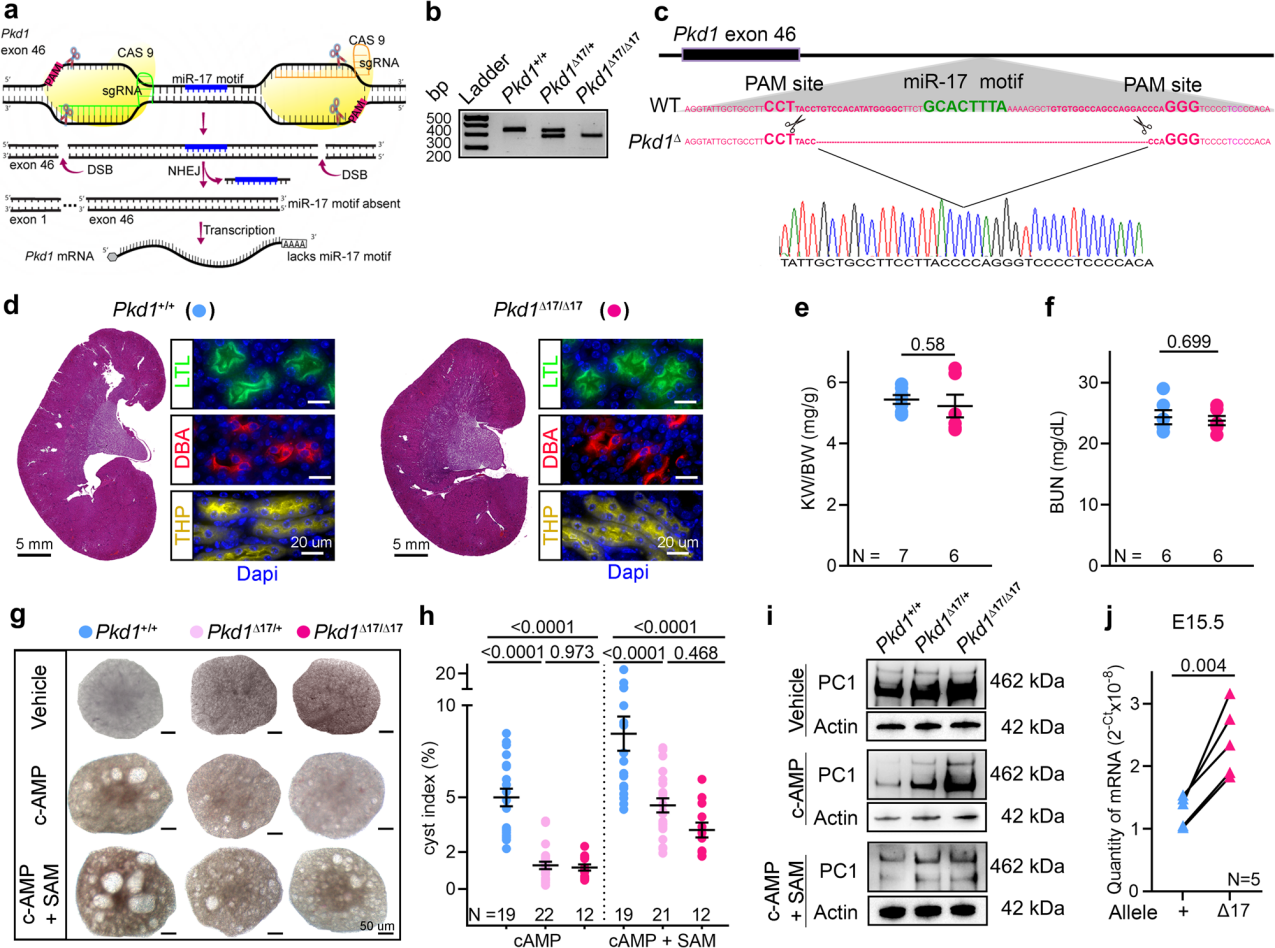
*Pkd1* mRNA is cis-repressed via its 3′-UTR miR-17 binding motif. **a** Graphic illustration of the CRISPR/Cas9 approach used to delete the miR-17 motif from *Pkd1* 3′-UTR (*Pkd1*^Δ17^). **b** PCR products obtained after amplification of tail DNA from mice with indicated genotypes. The lower band represents the Δ17 deletion. *n* = 3 for all genotypes. **c** 3′-UTR nucleotide sequence of wildtype (WT) and *Pkd1*^Δ17^ alleles. The miR-17 binding motif and sgRNA PAM sites are highlighted in bold green and pink, respectively. The pink dashed line indicates the deleted nucleotides in *Pkd1*^Δ17^. Sanger sequencing chromatogram depicting the nucleotide sequence of the *Pkd1*^Δ17^ allele is shown. **d** H&E staining, Lotus Tetragonolobus Lectin labeling (LTL, a proximal tubule marker), Tamm-Horsfall protein immunostaining (*THP*, a loop of Henle maker), and Dolichos Biflorus Agglutinin labeling (DBA, a collecting duct marker) showing normal kidney histology in 8-week-old *Pkd1*^+/+^ and *Pkd1*^Δ17/Δ17^ mice. **e–f** Normal kidney-weight-to-body-weight (KW/BW) and serum blood urea nitrogen (BUN) levels in 8-week-old *Pkd1*^+/+^ and *Pkd1*^Δ17/Δ17^ mice. **g–h** Images and cyst index quantification of E13.5 *Pkd1*^+/+^, *Pkd1*^Δ17/+^, and *Pkd1*^Δ17/Δ17^ kidneys grown for four days in culture media containing vehicle, 100 uM cAMP, or 100 uM cAMP plus 250 uM SAM. **i** Immunoblots depicting PC1 expression in *Pkd1*^+/+^, *Pkd1*^Δ17/+^, and *Pkd1*^Δ17/Δ17^ ex-vivo kidneys treated with vehicle, cAMP, or cAMP plus SAM. Actin is used as the loading control. **j** Allele-specific qRT-PCR showing the quantity of *Pkd1* mRNAs produced by the wildtype (+) and Δ17 alleles in E15.5 *Pkd1*^Δ17/+^ kidneys (*n* = 5). Error bars indicate SEM. Statistical analysis: Two-tailed Student’s *t*-test (**e**, **f**); One-way ANOVA, Tukey’s multiple-comparisons test (**h**); Two-tailed Paired *t*-test (**j**). Source data are provided as a Source Data file.

The impact of individual 3′-UTR MBEs on host gene regulation is mostly unstudied. We explored the idea that cis-repression via its 3′-UTR miR-17 binding motif is a novel mechanism governing *Pkd1* dosage. We began by examining the effect of deleting this MBE in normal mouse kidneys. We designed sgRNAs that bind to *Pkd1* exon-46, flanking the DNA segment that encodes the miR-17 motif, and used CRISPR/Cas9 editing to generate *Pkd1* alleles (*Pkd1*^∆17^) lacking the miR-17 binding site (Fig. 1a). First, we validated the motif deletion using DNA PCR followed by direct Sanger sequencing (Fig. 1b, c). Our editing approach did not inadvertently inactivate *Pkd1* since we observed normal kidney histology and renal function in 6-week-old and 18-week-old *Pkd1*^∆17/∆17^ mice (Fig. 1d–f and Supplementary Fig. 1). To assess PC1 levels, we performed western blot analysis using the 7E12 PC1 antibody, which detects the full-length protein and the n-terminal fragment. We first validated the 7E12 antibody in wildtype and *Pkd1*^*−/−*^ cells. As expected, we observed no PC1 signal in the *Pkd1*-null cells (Supplementary Fig. 2). Despite the loss of the miR-17 binding site, PC1 expression was the same between the kidneys of 6-week-old or 18-week-old *Pkd1*^∆17/∆17^ mice and their respective age-matched control *Pkd1*^+/+^ mice (Supplementary Fig. 1d), implying that there was no *Pkd1* cis-inhibition in the kidneys of normal adult mice.

Consistent with observations from others^27^, analysis of our miRNA microarray dataset revealed that miR-17 levels decline with postnatal maturation (Supplementary Fig. 3). Thus, the lack of *Pkd1* cis-inhibition in mature kidneys is likely due to the low basal miR-17 activity. We, therefore, switched to analyzing embryonic (E) *Pkd1*^∆17/+^ and *Pkd1*^∆17/∆17^ kidneys. We used ex vivo kidney organ culture to simultaneously assess the impact on *Pkd1* expression and cystogenesis. We cultured E13.5 littermate *Pkd1*^+/+^, *Pkd1*^∆17/+^, and *Pkd1*^∆17/∆17^ kidneys for four days in media containing 100 μM 8-bromo-cAMP or 100 μM 8-bromo-cAMP plus S-adenosylmethionine (SAM), or vehicle control (Fig. 1g, h). As expected^28^, cAMP increased cyst formation in *Pkd1*^+/+^ kidneys compared to vehicle treatment, and this effect was further enhanced with the addition of SAM. Interestingly, the pro-cystogenic effect of cAMP and SAM was attenuated in *Pkd1*^∆17/+^ and *Pkd1*^∆17/∆17^ kidneys (Fig. 1g, h). Moreover, we observed higher PC1 expression in *Pkd1*^∆17/+^ and *Pkd1*^∆17/∆17^ compared to *Pkd1*^+/+^ ex vivo cultured kidneys by immunoblot analysis (Fig. 1i). These data indicate that miR-17 motif deletion derepresses PC1 and blocks the pro-cystogenic effect of cAMP in embryonic cultured kidneys.

Next, we measured the relative abundance of wildtype and ∆17 transcripts within the total *Pkd1* mRNA pool. We designed allele-specific primers taking advantage of the unique mRNA sequence created by CRISPR/Cas9 editing of the ∆17 allele. qRT-PCR revealed that in E15.5 heterozygous in vivo *Pkd1*^∆17/+^ kidneys, the ∆17 allele contributed nearly 50% more transcripts than its wildtype counterpart (Fig. 1j). Similarly, we noted that the ∆17 allele produced more *Pkd1* mRNAs than the wildtype allele in ex vivo *Pkd1*^∆17/+^ kidney cultures. This difference became even more pronounced in the presence of cAMP (Supplementary Fig. 4a-c). These observations further imply inhibition of wildtype *Pkd1* mRNAs by miR-17 but an evasion of repression and improved stability of *Pkd1*^∆17^ mRNAs in embryonic kidneys.

### Endogenous monoallelic *Pkd1* derepression alleviates polycystic kidney disease

**Fig. 2:**
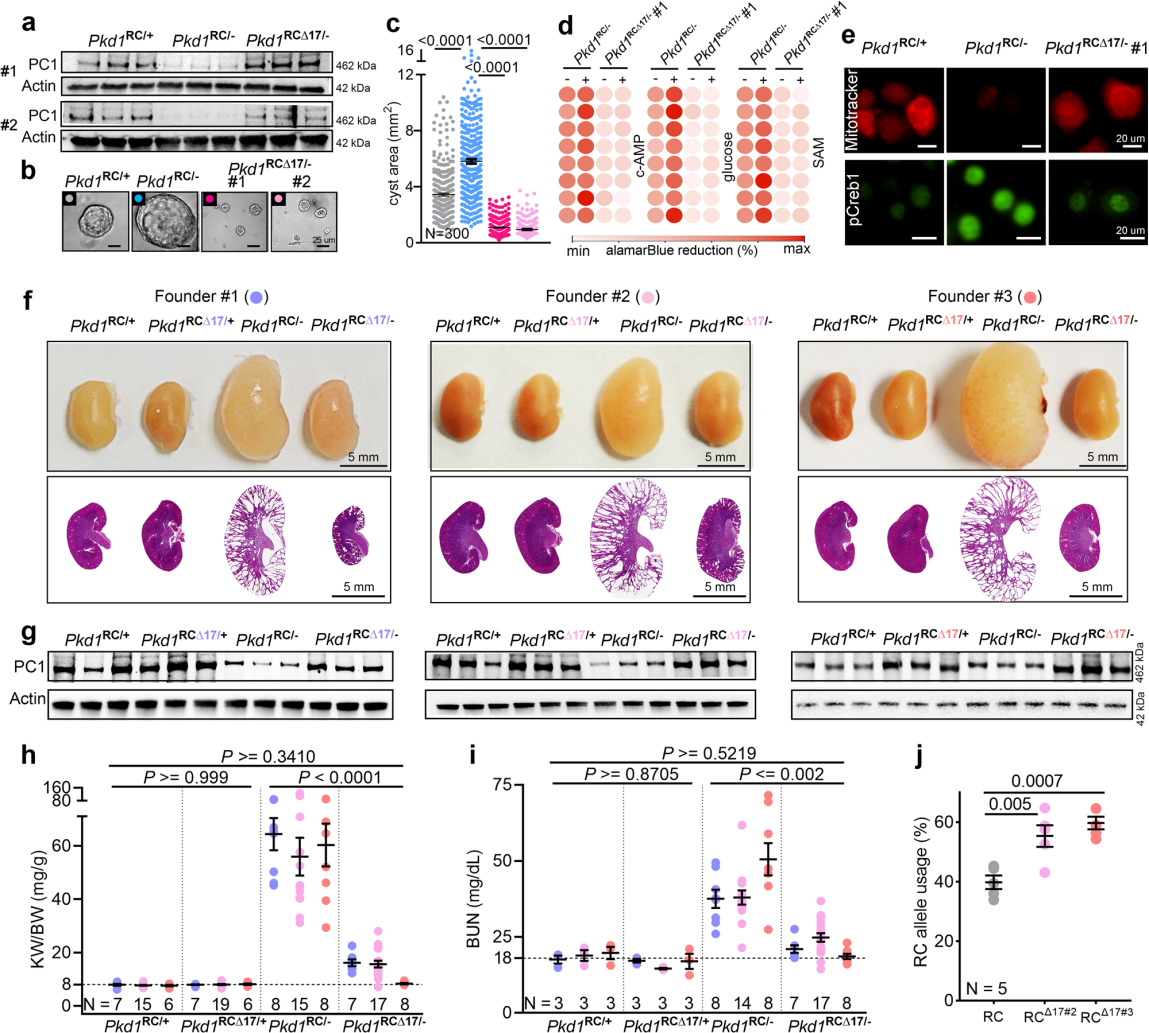
Monoallelic *Pkd1* derepression alleviates polycystic kidney disease. **a** Immunoblot showing reduced PC1 expression in *Pkd1*^RC/-^ compared to *Pkd1*^RC/+^ cells. PC1 level was restored in *Pkd1*^RCΔ17/-^ cells. #1 and #2 refer to the two independent *Pkd1*^RCΔ17/-^ clonal cell lines. Actin serves as the loading control. *n* = 3 biologically independent samples. **b–c** Representative images and quantification showing increased 3D cyst size of *Pkd1*^RC/-^ compared to *Pkd1*^RC/+^ cells cultured in Matrigel. Cyst size was normalized in *Pkd1*^RCΔ17/-^ cells. *n* = 300 cysts pooled from three independent experiments. **d** Heatmap showing alamarBlue-assessed proliferation of *Pkd1*^RC/-^ and *Pkd1*^RCΔ17/-^ cells in the absence (−) or presence (+) of 100 uM cAMP, 17 mM glucose, or 100 uM SAM. *n* = 8, each circle represents a biological replicate. **e** Representative images showing Mito-tracker labeling and anti-pCreb1 immunostaining in *Pkd1*^RC/+^, *Pkd1*^RC/-^, and *Pkd1*^RCΔ17/-^ cells. *n* = 3 biologically dependent experiments. **f** Gross kidney and H&E-stained kidney sections from 18-day-old mice with the indicated genotypes. Data from the progeny of the three founders are shown separately. Founder #1: *Pkd1*^RC/+^ (*n* = 7), *Pkd1*^RC∆17/+^ (*n* = 7), *Pkd1*^RC/-^ (*n* = 8), and *Pkd1*^RC∆17/-^ (*n* = 7). Founder #2: *Pkd1*^RC/+^ (*n* = 15), *Pkd1*^RC∆17/+^ (*n* = 19), *Pkd1*^RC/-^ (*n* = 15), and *Pkd1*^RC∆17/-^ (*n* = 17). Founder #3: *Pkd1*^RC/+^ (*n* = 6), *Pkd1*^RC∆17/+^ (*n* = 6), *Pkd1*^RC/-^ (*n* = 8), and *Pkd1*^RC∆17/-^ (*n* = 8). **g** Immunoblot showing PC1 expression in kidneys of 18-day-old mice with the indicated genotypes derived from the three founders. Actin serves as the loading control. *n* = 3 independent kidney samples for each genotype and founder. **h**, **i** KW/BW ratio and BUN levels in mice with the indicated genotypes are shown. Data from all three founders are shown. Founder#1 (blue circles), Founder#2 (light pink circles), and Founder#3 (orange circles). (**j**) Paired-end RNA-seq data showing the RC allele usage in *Pkd1*^RC/-^ (grey circles, *n* = 5), *Pkd1*^RCΔ17/-^ founder#2 (pink circles, *n* = 5), and *Pkd1*^RCΔ17/-^ founder#3 (orange circles, *n* = 5). Error bars indicate SEM. Statistical analysis: One-way ANOVA, Tukey’s multiple-comparisons test (**c**, **h**–**j**). Source data are provided as a Source Data file.

Kidney cyst formation ensues when *PKD1* dosage falls below a critical threshold. However, no approach exists to reverse the *PKD1* decline. Our observations in normal embryonic kidneys prompted us to ask whether *Pkd1* is cis-repressed in ADPKD and if preventing this inhibition has a disease-modifying impact. We first examined the *Pkd1*^RC/−^ cellular ADPKD model. This is a collecting duct-derived mouse cell line that harbors the missense RC mutation on one *Pkd1* allele, whereas the other allele is inactivated^29^. The RC mutation results in arginine to cystine substitution two amino acids before the second transmembrane domain and reduces mature (functional) PC1 protein levels. The RC mutation maps to *Pkd1* exon-30 and is significantly upstream of the miR-17 motif, which is encoded by *Pkd1* exon-46. This allowed us to use CRISPR/Cas9 editing to remove the 3′-UTR miR-17 motif from the RC allele (*Pkd1*^RC∆17/−^) (Supplementary Fig. 5). We generated two independent *Pkd1*^RC∆17/−^ clonal cell lines and characterized them both in relation to the unedited parental *Pkd1*^RC/-^ and *Pkd1*^RC/+^ cells. We previously reported that PC1 expression was reduced in *Pkd1*^RC/-^ cells compared to *Pkd1*^RC/+^ cells. Remarkably, western blot analysis using the 7E12 antibody revealed that eliminating the miR-17 motif restored PC1 expression in *Pkd1*^RC∆17/-^ cell lines (Fig. 2a and Supplementary Fig. 7a). Next, employing several independent assays, we demonstrated that this degree of PC1 derepression was sufficient to reverse several well-known pathogenic events linked to cyst growth. First, we noted a higher proliferation rate and 3D cyst size in *Pkd1*^RC/-^ cells than in *Pkd1*^RC/+^ cells, which were normalized after PC1 derepression in *Pkd1*^RC∆17/-^ cells (Fig. 2b, c). Second, we observed that while cAMP, glucose, and SAM increased the already elevated proliferation of *Pkd1*^RC/-^ cells, *Pkd1*^RC∆17/-^ cells were resistant to these pro-proliferative stimuli (Fig. 2d and Supplementary Table 1). Third, we used MitoTracker to assess mitochondrial membrane potential as a proxy of oxidative phosphorylation and anti-pCreb1 antibody immunofluorescence as a readout of c-AMP signaling. Compared to *Pkd1*^RC/+^ cells, we observed a reduced MitoTracker signal and higher pCreb1 expression in *Pkd1*^RC/-^ cells. The opposite was true for *Pkd1*^RC∆17/-^ cells, which exhibited restored MitoTracker signal and lowered pCreb1 expression (Fig. 2e and Supplementary Fig. 6b). Finally, immunoblot analysis revealed elevated Yap1, pCreb1, and c-Myc expression in *Pkd1*^RC/-^ cells compared to *Pkd1*^RC/+^ cells, which returned to baseline in *Pkd1*^RC∆17/-^ cells (Supplementary Fig. 6c).

**Fig. 3:**
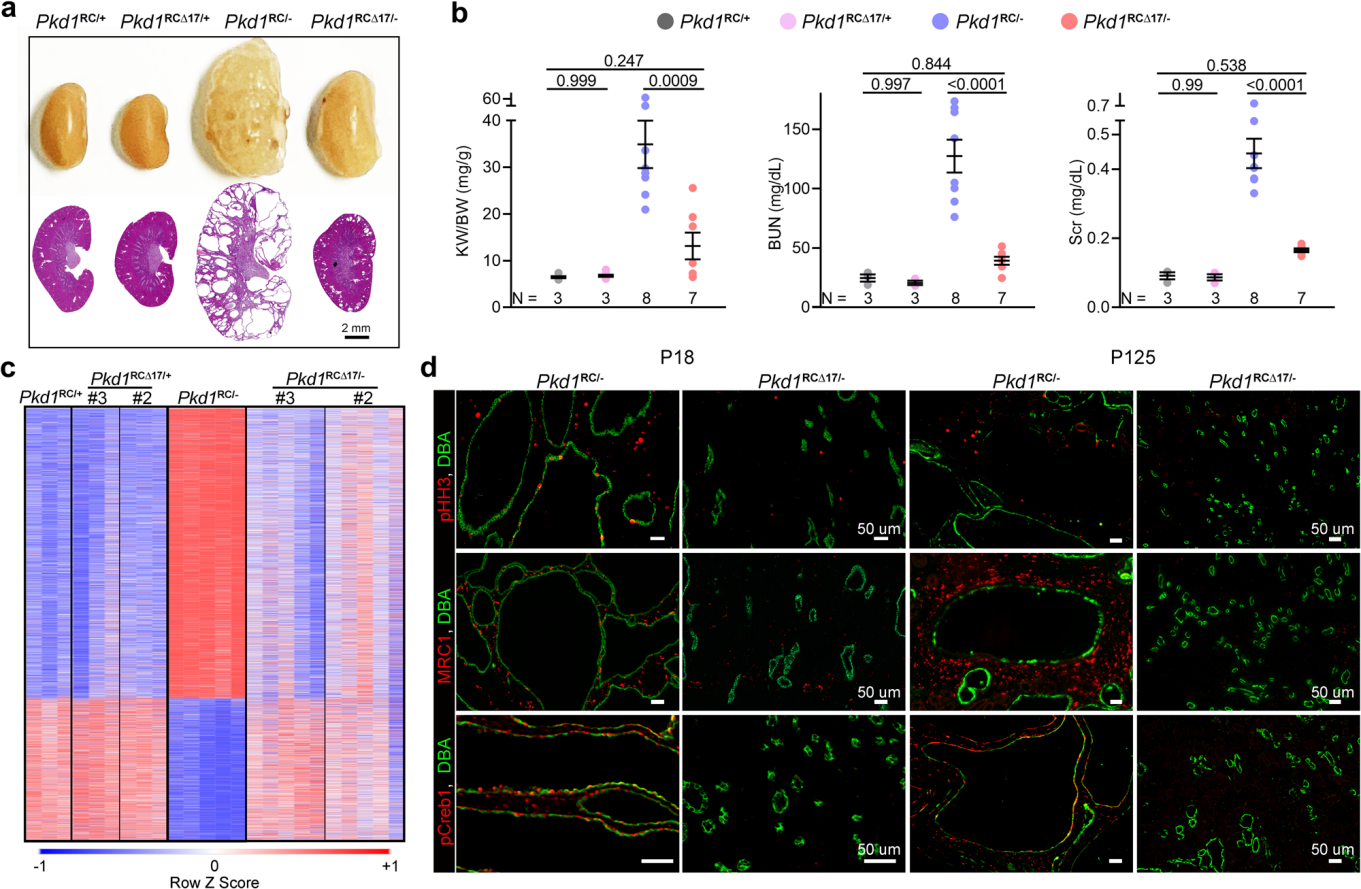
*Pkd1* derepression attenuates cyst-pathogenic events and disease progression. **a** Gross kidney images and H&E-stained kidney sections from 18-week-old mice with the indicated genotypes derived from founder#3. miR-17 motif deletion was associated with sustained benefit and suppressed long-term PKD progression. *n* = 3 (*Pkd1*^RC/+^), *n* = 3 (*Pkd1*^RC∆17/+^), *n* = 8 (*Pkd1*^RC/-^), and *n* = 7 (*Pkd1*^RC∆17/-^). **b** KW/BW, BUN, and serum creatinine (Scr) levels in the 18-week-old progeny of founder#3 are shown. **c** Heatmap showing global mRNA expression profiles of kidneys from 18-day-old mice with the indicated genotypes. mRNAs that were dysregulated in *Pkd1*^RC/-^ compared to *Pkd1*^RC/+^ kidneys but exhibited improved expression in *Pkd1*^RCΔ17/-^ kidneys were chosen for visualization. #3 = founder#3; #2 = founder#2. *n* = 3 (*Pkd1*^RC/+^), *n* = 3 (*Pkd1*^RC∆17/+^ founder 2), *n* = 3 (*Pkd1*^RC∆17/+^ founder 3), *n* = 5 (*Pkd1*^RC/-^), *n* = 5 (*Pkd1*^RC∆17/-^ founder 2), and *n* = 5 (*Pkd1*^RC∆17/-^ founder 3). (**d**) Representative images showing phospho-Histone-H3 (pHH3), Mannose Receptor C-Type 1 (MRC1), or pCreb1 immunostaining in kidney sections of 18-day-old and 18-week-old *Pkd1*^RC/-^ (*n* = 5) and *Pkd1*^RCΔ17/-^ (*n* = 5) mice. The sections were co-labeled with DBA to mark collecting duct-derived cysts. Error bars indicate SEM. Statistical analysis: One-way ANOVA, Tukey’s multiple-comparisons test (**b**). Source data are provided as a Source Data file.

Based on these encouraging results, we next modeled the 3′-UTR ∆17 deletions in vivo. We CRISPR-edited Ksp^Cre+^; *Pkd1*^RC/RC^ fertilized eggs to eliminate the miR-17 motif from the *Pkd1* RC allele. We implanted these eggs into pseudopregnant surrogate female mice and eventually obtained three germline-transmitting heterozygous Ksp^Cre+^; *Pkd1*^RC∆17/RC^ founder mice. Using DNA PCR and Sanger sequencing, we validated that the miR-17 motif was indeed deleted in all three founder mice from one RC allele, whereas the other RC allele still contained the wildtype 3′-UTR (Supplementary Fig. 8). We selected mice with heterozygous ∆17 deletions because breeding them with *Pkd1*^F/F^ mice allowed us to generate the following four relevant genotypes from the same breeding pair: *Pkd1*^RC/F^ (*Pkd1*^RC/+^), *Pkd1*^RC∆17/F^ (*Pkd1*^RC∆17/+^), Ksp^Cre+^; *Pkd1*^RC/F^ (*Pkd1*^RC/-^), and Ksp^Cre+^; *Pkd1*^RC∆17/F^ (*Pkd1*^RC∆17/-^). Data from the 18-day-old progeny of all three founders are shown in Fig. 2F–H. First, we noted normal kidney histology and function in *Pkd1*^RC∆17/+^ mice, again indicating that miR-17 motif deletion does not disrupt *Pkd1* or produce PKD (Fig. 2f). For each founder progeny, we observed reduced PC1 expression, severe cystic kidney disease, an increased kidney-weight-to-body-weight (KW/BW) ratio, and higher serum BUN levels in *Pkd1*^RC/-^ mice than in *Pkd1*^RC/+^ mice (Fig. 2f–j). As with the cell lines, miR-17 motif deletion caused PC1 derepression, as assessed using the 7E12 antibody, in *Pkd1*^RC∆17/-^ kidneys compared to *Pkd1*^RC/-^ kidneys (Fig. 2g). We verified PC1 derepression in cell lines and mice using a second independent antibody generated by the U Maryland PKD center (see methods for details) that detects the PC1 c-terminus (Supplementary Fig. 7). Moreover, using paired-end RNA-seq, we noted higher RC allele usage in *Pkd1*^RC∆17/-^ kidneys than in *Pkd1*^RC/-^ kidneys, further indicating *Pkd1* derepression (Fig. 2j). Strikingly, the cystic disease was almost completely alleviated, and KW/BW and serum BUN were nearly normalized in *Pkd1*^RC∆17/-mice^ compared to *Pkd1*^RC/-^ mice (Fig. 2f–i). To examine the long-term effects, we prospectively followed founder #2 and #3 progeny for 8 and 18 weeks, respectively. Founder#2 progeny exhibited an aggressive cystic disease phenotype, with 76.4% (13/17) of *Pkd1*^RC/-^ mice succumbing to kidney failure before eight weeks of age (Supplementary Fig. 9a, b). Moreover, the four surviving *Pkd1*^RC/-^ mice exhibited severe PKD and near-fatal kidney failure. In contrast, only 27.2% (6/22) of *Pkd1*^RC∆17/-^ mice died by eight weeks, and the surviving mice had fewer cysts and relatively preserved kidney function (Supplementary Fig. 9c-e). All founder#3 *Pkd1*^RC/-^ progeny survived until 18 weeks of age. However, they developed progressive kidney failure, as evidenced by an average blood urea nitrogen (BUN) of >100 mg/dl and serum creatinine of >0.4 mg/dl (Fig. 3b). Founder #3 *Pkd1*^RC∆17/-^ mice exhibited minimal disease progression with average BUN < 30 mg/dl and serum creatinine <0.2 mg/dl (Fig. 3a, b).

Large-scale transcriptomic dysregulation, activation of tubular proliferation and oncogenic signaling, and interstitial inflammation are some of the key pathological hallmarks of ADPKD. We next addressed whether these changes were blunted by PC1 derepression. We performed RNA-seq analysis using kidney samples from 18-day-old *Pkd1*^RC/+^, *Pkd1*^RC∆17/+^, *Pkd1*^RC/-^, and *Pkd1*^RC∆17/-^ mice. We observed dysregulation of an extensive network of gene transcripts with upregulation of 4157 and downregulation of 2067 mRNAs in cystic *Pkd1*^RC/-^ compared to noncystic *Pkd1*^RC/+^ control kidneys (Fig. 3c). Mirroring kidney histology, *Pkd1*^RC∆17/+^ exhibited a nearly identical gene expression pattern as *Pkd1*^RC/+^ kidney. Impressively, >95% of dysregulated mRNAs in *Pkd1*^RC/-^ kidneys showed improved (or normalized) expression in *Pkd1*^RC∆17/-^ kidneys (Fig. 3c). Consistent with the RNA-seq data, immunoblot analysis revealed reduced c-Myc and Yap1 in the kidneys of 18-day-old *Pkd1*^RC∆17/-^ mice compared to *Pkd1*^RC/-^ mice (Supplementary Fig. 9f). Finally, immunofluorescence analysis demonstrated fewer anti-phospho-Histone-H3-positive cells, indicating lower proliferation, and reduced anti-pCreb1 and anti-MRC1 signals, implying attenuated c-AMP signaling and cyst-associated inflammation, respectively, in kidneys of 18-day-old and 18-week-old *Pkd1*^RC∆17/-^ mice compared to *Pkd1*^RC/-^ mice (Fig. 3d).

### Preventing *Pkd2* cis-inhibition attenuates cyst growth in *Pkd1*-mutant models

**Fig. 4:**
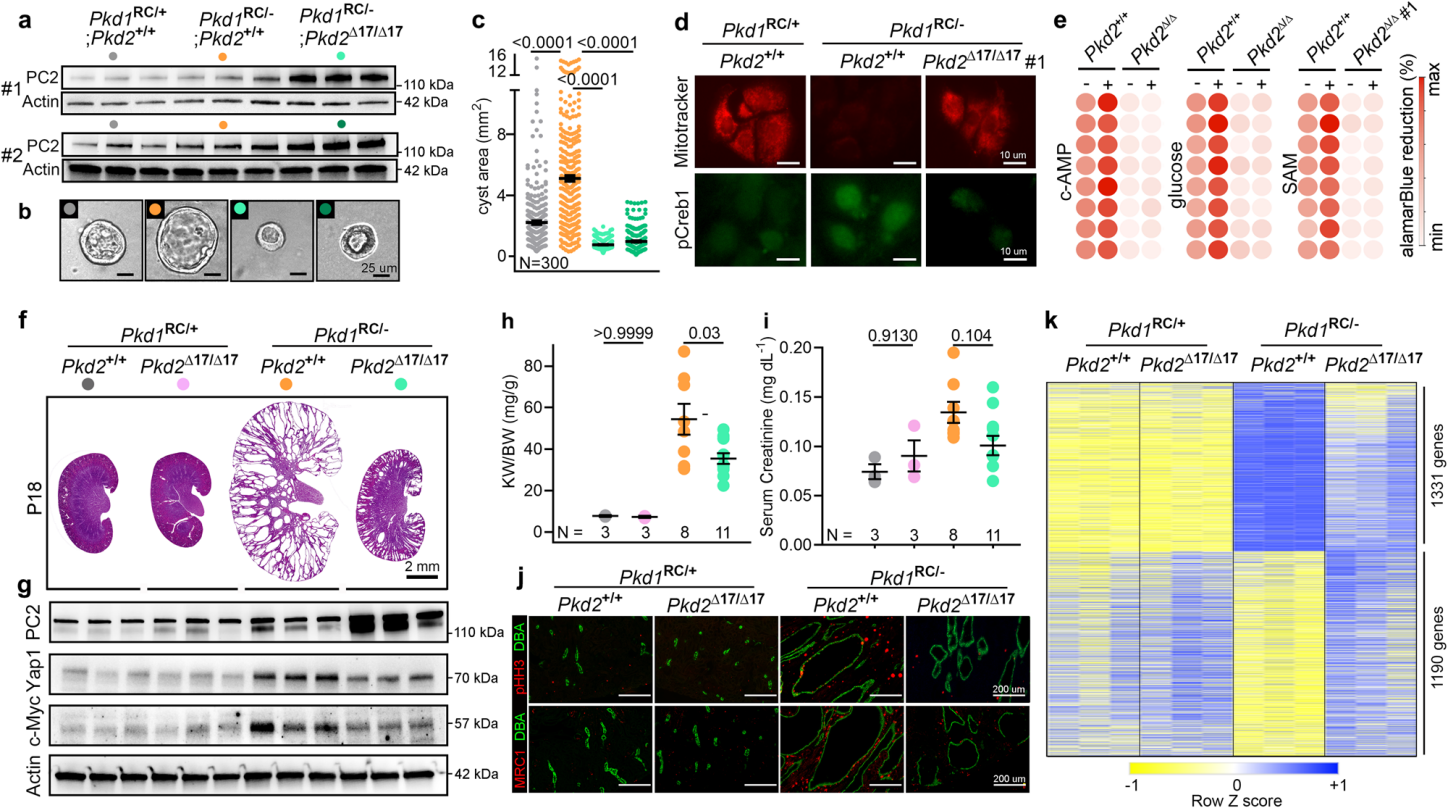
*Pkd2* derepression retards cyst growth in *Pkd1*-mutant models. **a** Immunoblot showing Polycystin-2 (PC2) expression in cells with the indicated genotypes. miR-17 motif deletion from *Pkd2* 3′-UTR leads to higher PC2 expression in *Pkd1*^RC/-^ cells. Actin serves as the loading control. #1 and #2 refer to the two independent *Pkd1*^RC/-^; *Pkd2*^Δ17/Δ17^ clonal cell lines*. n* = 3 biologically independent samples for both clones. **b**, **c** Representative images and quantification showing 3D cyst size of cells with indicated genotypes grown in Matrigel cultures. *n* = 300 cysts pooled from three independent experiments. **d** Representative images showing mitotracker labeling and anti-pCreb1 immunostaining in cells with the indicated genotypes. *n* = 3 biologically independent experiments. **e** Heatmap showing alamarBlue-assessed proliferation of *Pkd1*^RC/-^ and *Pkd1*^RC/-^; *Pkd2*^Δ17/Δ17^ cells in the absence (−) or presence (+) of cAMP, glucose, or SAM. *n* = 8, each circle represents a biological replicate. **f** H&E-stained kidney sections from 18-day-old mice with the indicated genotypes are shown. *n* = 3 (*Pkd1*^RC/+^; *Pkd2*^*+/+*^), *n* = 3 (*Pkd1*^RC/+^; *Pkd2*^∆17/∆17^), *n* = 8 *Pkd1*^RC/-^; *Pkd2*^*+/+*^), and *n* = 11 (*Pkd1*^RC/-^; *Pkd2*^∆17/∆17^). **g** Immunoblots showing PC2, Yap1, and c-Myc expression in kidneys of 18-day-old mice with the indicated genotypes (*n* = 3 for each group). **h**, **i** KW/BW and serum creatinine levels in 18-day-old mice with the indicated genotypes. **j** Representative images showing pHH3 and MRC1 immunostaining in kidney sections from 18-day-old mice with the indicated genotypes (*n* = 3 for each group). **k** Heatmap showing differential mRNA expression in kidneys of 18-day-old mice with the indicated genotypes (*n* = 3). mRNAs that were dysregulated in *Pkd1*^RC/-^ compared to *Pkd1*^RC/+^ kidneys but exhibited improved expression in *Pkd1*^RC/-^*; Pkd2*^Δ17/Δ17^ kidney were chosen for heatmap visualization. Error bars indicate SEM. Statistical analysis: One-way ANOVA, Tukey’s multiple-comparisons test (b, h, and i). Source data are provided as a Source Data file.

A long-standing question has been whether increasing PC2 can compensate for low PC1. Interestingly, similar to *PKD1*, *PKD2* harbors an evolutionarily conserved 3′-UTR miR-17 motif. Our work indicates heightened miR-17 repressive activity in *Pkd1*-mutant ADPKD models. Therefore, we wondered whether *Pkd2* is cis-inhibited and whether preventing this autoinhibition positively impacts disease progression in *Pkd1*-mutant models. To address these questions, we again began with the *Pkd1*^*RC/-*^ cellular model, but this time used CRISPR/Cas9 to delete the miR-17 motifs from the *Pkd2* 3′-UTR (*Pkd1*^RC/-^; *Pkd2*^∆17/∆17^) while keeping the *Pkd1* miR-17 motif intact (Supplementary Fig. 10). Consistent with 3′-UTR cis-inhibition, using qRT-PCR and immunoblot analysis, we observed higher *Pkd2* and PC2 expression in two *Pkd1*^RC/-^; *Pkd2*^∆17/∆17^ clonal cell lines compared to their unedited parental *Pkd1*^RC/*-*^ cells (Fig. 4a). PC1 expression remained unchanged between the edited and unedited cells, indicating the specificity of miR-17 motif deletion from *Pkd2* 3′-UTRs (Supplementary Fig. 11b). Surprisingly, we noted that PC2 derepression was associated with reduced 3D cyst growth, restored MitoTracker signal, and downregulation of pCreb1, Yap1, Mettl3, and c-Myc expression in *Pkd1*^RC/-^; *Pkd2*^∆17/∆17^ cells compared to *Pkd1*^RC/-^ cells (Fig. 4b-d and Supplementary Fig. 11b-c). As was the case with the *Pkd1* 3′-UTR deletions, while cAMP, glucose, and SAM promoted proliferation of *Pkd1*^*RC/-*^ cells, we noted that this stimulatory effect was lost in *Pkd1*^*RC/-*^; *Pkd2*^∆17/∆17^ cells (Fig. 4e, Supplementary Table 2).

Our observations in *Pkd1*^RC/-^ cells suggest a tantalizing possibility that preventing *Pkd2* cis-inhibition and improving PC2 expression compensates and retards disease progression in *Pkd1*-mutant models. We tested this notion in vivo by deleting the *Pkd2* 3′-UTR miR-17 motif (*Pkd2*^∆17/∆17^) in *Pkd1*^RC/-^ mice. Briefly, we CRISPR/Cas9-edited Ksp^Cre^; *Pkd1*^RC/RC^ mice to generate Ksp^Cre^; *Pkd1*^RC/RC^; *Pkd2*^∆17/+^ mice (see Supplementary Fig. 12 for details). These mice were then bred with *Pkd1*^F/F^ mice to eventually generate the following four genotypes: (i) *Pkd1*^RC/F^; *Pkd2*^*+/+*^, (ii) *Pkd1*^RC/F^; *Pkd2*^∆17/∆17^, (iii) Ksp^Cre^; *Pkd1*^RC/F^; *Pkd2*^*+/+*^, and (iv) Ksp^Cre^; *Pkd1*^RC/F^; *Pkd2*^∆17/∆17^. Characterization of these mice revealed that *Pkd2* miR-17 motif deletion in the noncystic setting did not cause PC2 upregulation, and both *Pkd1*^RC/F^; *Pkd2*^*+/+*^ and *Pkd1*^RC/F^; *Pkd2*^∆17/∆17^ mice exhibited normal kidney histology and function (Fig. 4f, g). In contrast, *Pkd2* miR-17 motif deletion in cystic *Pkd1*^RC/-^ mice was associated with higher PC2 expression. Moreover, KW/BW and serum creatinine levels were reduced by 34.8 and 25%, respectively, in *Pkd1*^RC/-^; *Pkd2*^∆17/∆17^ compared to *Pkd1*^RC/-^; *Pkd2*^*+/+*^ mice (Fig. 4h, i). Consistently, we observed that compared to *Pkd1*^RC/-^; *Pkd2*^*+/+*^ kidneys, *Pkd1*^RC/-^; *Pkd2*^∆17/∆17^ kidneys exhibited reduced c-Myc and Yap1 expression (Fig. 4G) and lower cyst proliferation and interstitial inflammation (Fig. 4j). As an additional phenotypic characterization, we performed RNA-seq analysis to compare the kidney transcriptomic profile in the four groups of mice (Fig. 4k). The mRNA expression patterns were nearly identical in *Pkd1*^RC/F^; *Pkd2*^*+/+*^ and *Pkd1*^RC/F^; *Pkd2*^∆17/∆17,^ further implying that *Pkd2* miR-17 motif elimination has minimal impact in noncystic kidneys. As expected, the cystic *Pkd1*^RC/-^; *Pkd2*^*+/+*^ kidneys exhibited widespread mRNA dysregulation compared to noncystic *Pkd1*^RC/+^; *Pkd2*^*+/+*^ control kidneys. We found that *Pkd2* miR-17 motif deletion was associated with improved expression of nearly 50% of these dysregulated mRNAs in *Pkd1*^RC/-^; *Pkd2*^∆17/∆17^ (Fig. 4k).

### Acute blockade of *Pkd1* and *Pkd2* cis-inhibition ameliorates PKD

**Fig. 5:**
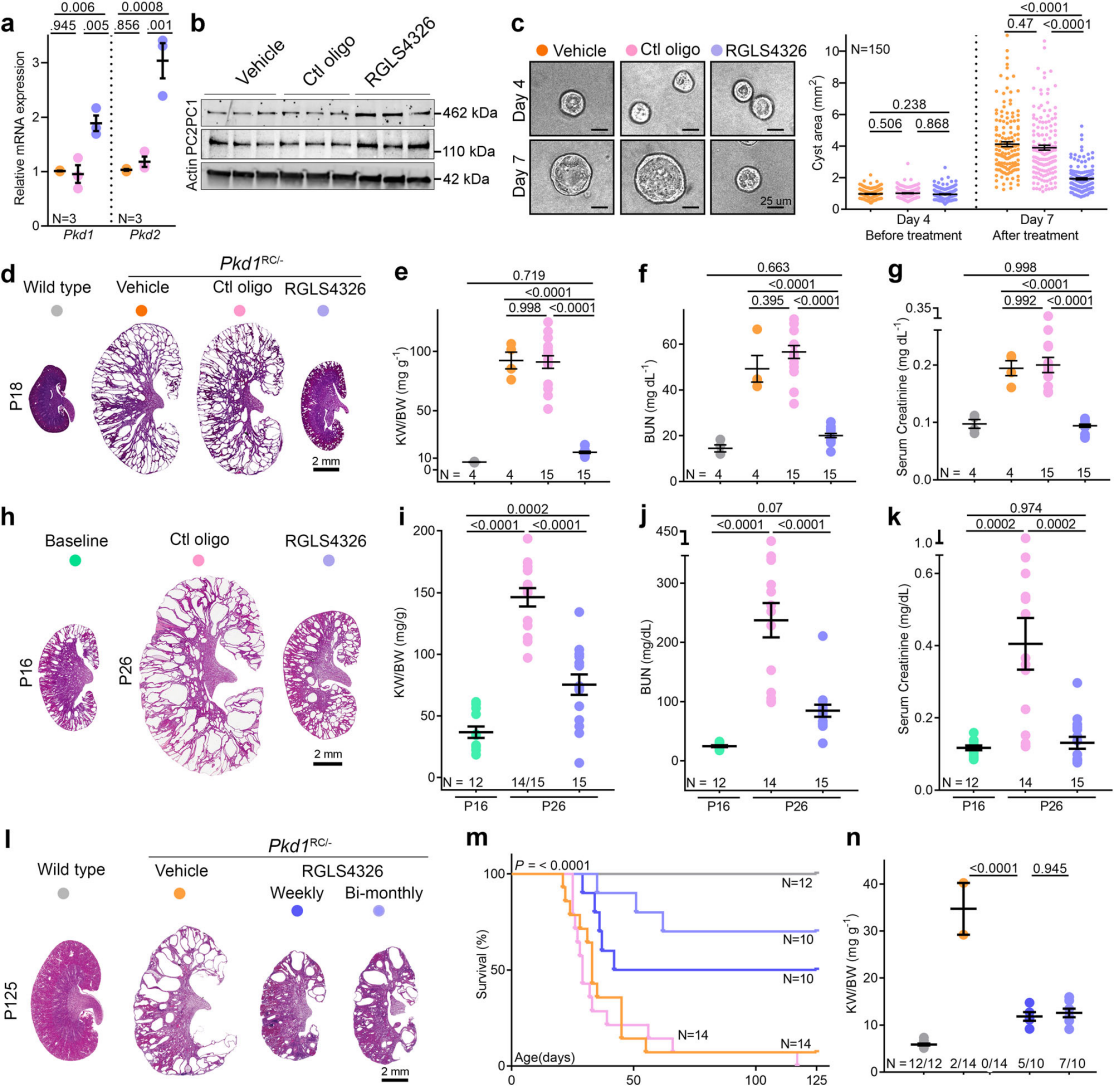
Acute *Pkd1* and *Pkd2* derepression attenuate PKD. **a–b** qRT-PCR and immunoblot analysis showing *Pkd1*/PC1 and *Pkd2*/PC2 expression in *Pkd1*^RC/-^ cells transfected with vehicle (PBS), 100 uM control oligonucleotide, or 100 uM RGLS4326. **c** Images and quantification of 3D cyst size of *Pkd1*^RC/-^ cells cultured in Matrigel before (day 4) or after (day 7) transfection with vehicle (PBS), 100 uM control oligonucleotide, or 100 uM RGLS4326. **d** H&E-stained kidney sections from 18-day-old *Pkd1*^RC/-^ mice injected on P10, P11, P12, and P16 either with vehicle (PBS), 20 mg/kg control oligonucleotide, or 20 mg/kg RGLS4326. H&E-stained kidney section from untreated 18-day-old wildtype mouse is shown for reference. **e–g** KW/BW, BUN, and serum creatinine levels in 18-day-old *Pkd1*^RC/-^ mice treated with vehicle (PBS), 20 mg/kg control oligonucleotide, or 20 mg/kg RGLS4326 are shown. Data from untreated 18-day-old wildtype mice are shown as a reference. **h** H&E-stained kidney sections from 26-day-old *Pkd1*^RC/-^ mice injected on P16 and P17 with 20 mg/kg control oligonucleotide or 20 mg/kg RGLS4326. H&E-stained kidney sections from genetically matched but untreated 16-day-old *Pkd1*^RC/-^ mice are shown to depict disease prior to starting treatment. **i–k** KW/BW, BUN, and serum creatinine levels in untreated 16-day-old or treated 26-day-old *Pkd1*^RC/-^ mice are shown. **l–n** *Pkd1*^RC/-^ mice were injected on P16 and P17 with vehicle, 20 mg/kg RGLS4326, or 20 mg/kg control oligonucleotide. These mice then received their respective treatment regimen every week until 18 weeks of age. The fourth cohort of *Pkd1*^RC/-^ mice received 20 mg/kg RGLS4326 treatment on P16, P17, and bimonthly thereafter. **l** H&E-stained kidney sections of 125-day-old *Pkd1*^RC/-^ mice on the vehicle or RGLS4326 treatment are shown. **m** Kaplan-Meir survival curves of *Pkd1*^RC/-^ mice in the four treatment groups. Survival of untreated wildtype is shown as a reference. **n** KW/BW ratio in mice that survived till 125 days. Wildtype mice: *n* = 3; *Pkd1*^RC/-^ mice: *n* = 2 (vehicle treatment), *n* = 5 (weekly RGLS4326 treatment), and *n* = 7 (bi-monthly RGLS4326 treatment). Error bars indicate SEM. Statistical analysis: One-way ANOVA, Tukey’s multiple-comparisons test (**a**, **c**, **e–g**, **i–k**, **n**); Mantel-Cox (**m**). Source data are provided as a Source Data file.

Our CRISPR-edited clonal cellular or mouse ADPKD models lead to chronic *Pkd1* or *Pkd2* derepression. Therefore, we could not assess whether acute derepression of *Pkd1*/*2*, just as the cysts are forming, will prevent disease onset or if restoring *Pkd1*/*2* can reign in established PKD. To answer these questions, we employed the anti-miR-17 oligonucleotide RGLS4326 as a tool to acutely block *Pkd1* and *Pkd2* cis-inhibition^30^. First, we confirmed that compared to vehicle (PBS) or control oligonucleotide, RGLS4326 increased *Pkd1/2* and PC1/2 expression in *Pkd1*^RC/-^ cells (Fig. 5a, b). The *Pkd1/2-*boosting effect of RGLS4326 was apparent within three days after treatment. Importantly, RGLS4326 treatment did not lead to higher PC1 and PC2 levels in *Pkd1*^RC∆17/-^ and *Pkd1*^RC/-^; *Pkd2*^∆17/∆17^ cell lines, respectively, confirming that the upregulation of polycystins by this oligonucleotide relies on the miR-17 motif in *Pkd1/2* 3′-UTRs (Supplementary Fig. 14b-c). RGLS4326-treated *Pkd1*^RC/-^ cells had reduced proliferation, produced smaller cysts in 3D Matrigel cultures, exhibited lower Yap1, c-Myc, and pCreb1 expression, and a higher MitoTracker signal compared to PBS- or control oligonucleotide-treated *Pkd1*^RC/-^ cells (Supplementary Fig. 13). The cyst-reducing effect of RGLS4326 was present but blunted in *Pkd1*^RC∆17/-^ or *Pkd1*^RC/-^; *Pkd2*^∆17/∆17^ cell lines, suggesting that this compound mediates its benefits in *Pkd1*^RC/-^ cells primarily via *Pkd1/2* derepression (Supplementary Fig. 14d-g). Next, we tested the impact of acutely raising *Pkd1/2* in *Pkd1*^RC/-^ cells after cysts had already formed. We cultured untreated *Pkd1*^RC/-^ cells in Matrigel for four days, allowing the cyst to grow. We then treated these cysts with a vehicle, control oligonucleotide, or RGLS4326, and monitored them for three additional days. Vehicle and control oligonucleotide-treated cysts nearly tripled in size, whereas RGLS4326 treatment suppressed this growth (Fig. 5c).

We next examined whether the observations in cells can be replicated in vivo. In the first study, we treated *Pkd1*^RC/-^ mice with vehicle (PBS), control oligonucleotide, or RGLS4326 starting at P10, the age at which cysts begin to form in this model. By P18, we noted marked kidney enlargement with a > 10-fold higher KW/BW ratio and elevated BUN and serum creatinine in PBS and control oligonucleotide-treated mice compared to age-matched wildtype mice (Fig. 5d–g). Strikingly, PKD was virtually prevented, and renal function remained normal in P18 RGLS4326-treated *Pkd1*^RC/-^ mice (Fig. 5d–g). In the second study, we began treatment at P16 when *Pkd1*^RC/-^ mice had already developed cystic disease. By P26, one out of 15 control oligonucleotide-treated mice had died, and the surviving mice had developed progressive kidney enlargement and near-fatal kidney failure. In contrast, we observed attenuation of PKD progression and stabilization of kidney function in RGLS4326-treated *Pkd1*^RC/*-*^-KO mice (Fig. 5h–k). Finally, in a third study, we assessed the long-term effects of *Pkd1/2* derepression in mice that had already developed PKD. We treated *Pkd1*^RC/-^ mice on P16 and P17 with the vehicle, 20 mg/kg RGLS4326, or 20 mg/kg control oligonucleotide. These mice then received their respective treatment regimens every week until 18 weeks of age. A fourth group of *Pkd1*^RC/-^ mice received 20 mg/kg RGLS4326 treatment on P16 and P17 and every other week thereafter. 85.7% (12 out of 14) of PBS-treated and 100% (14 out of 14) of control oligonucleotide-treated *Pkd1*^RC/-^-KO mice succumbed to their disease before 18 weeks of age. In contrast, 70% (7 out of 10) and 50% (5 out of 10) of *Pkd1*^RC/-^ mice treated with RGLS4326 bi-monthly or weekly, respectively, survived until 18 weeks of age (Fig. 5m). Furthermore, we noted substantially preserved kidney parenchyma (Fig. 5l and Supplementary Fig. 15) and reduced KW/BW (Fig. 5n) among the surviving mice in the RGLS4326 group. Thus, acute pharmaceutical *Pkd1/2* derepression, including after cyst onset, attenuates murine PKD.

### *PKD1*^∆17^ or *PKD2*^∆17^ alleles reduce cyst growth of patient-derived primary ADPKD cultures

**Fig. 6:**
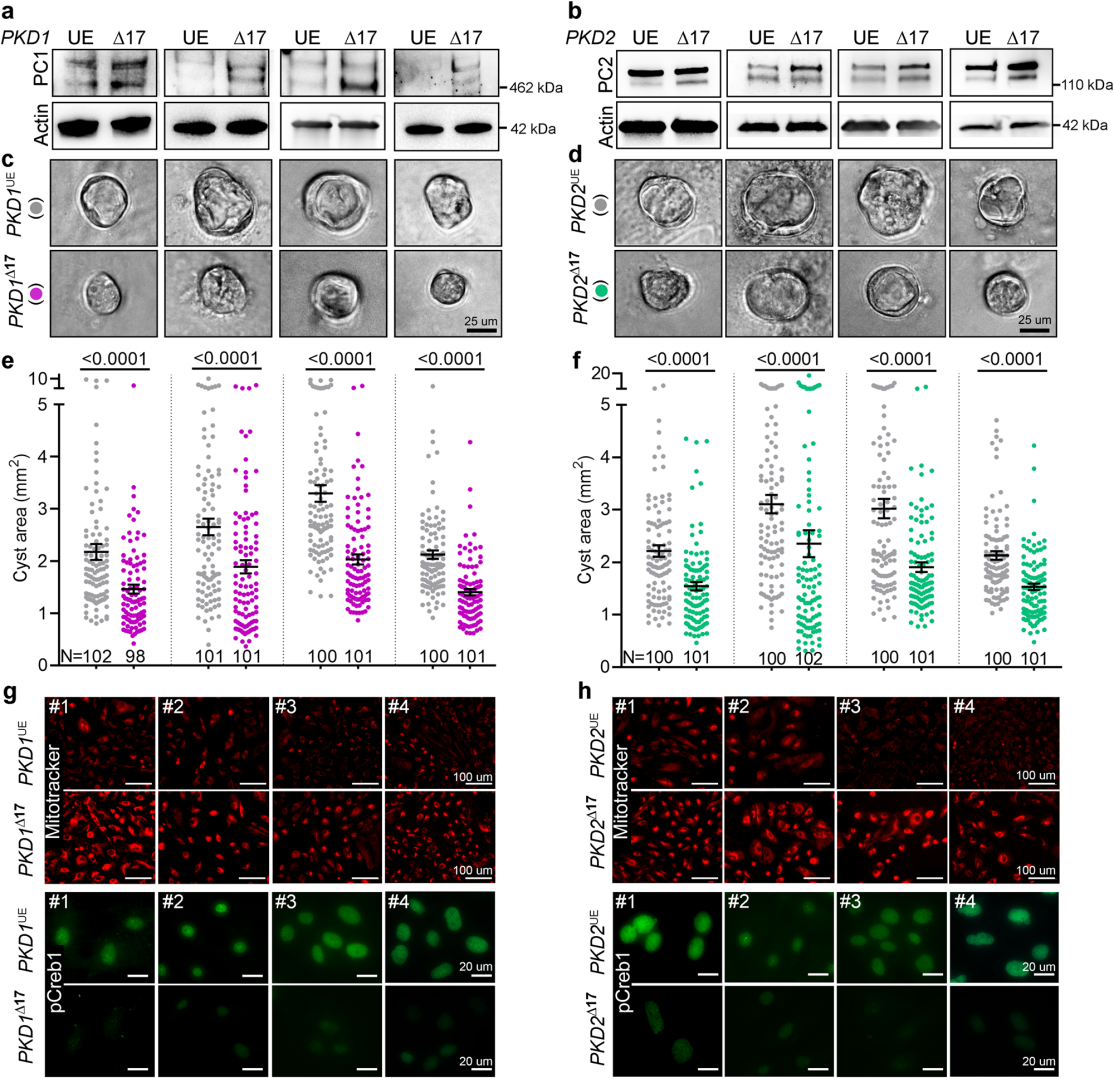
*PKD1*^Δ17^ or *PKD2*^Δ17^ reduce 3D cyst growth in primary human ADPKD cultures. CRISPR/Cas9-editing was used to delete the miR-17 motif from *PKD1* 3′-UTR (*PKD1*^Δ17^) or *PKD2* 3′-UTR (*PKD2*^Δ17^) in primary ADPKD cultures from four human donors (#1 through #4). **a**, **b** Immunoblots showing higher PC1 expression in *PKD1*^Δ17^ and higher PC2 expression in *PKD2*^Δ17^ ADPKD cultures compared to their respective unedited (UE) parental ADPKD cultures. Protein bands are 460 kDa (**a**) and 110–120 kDa size (**b**). Actin serves as the loading control. **c–f** Images and quantification showing reduced cyst size of *PKD1*^Δ17^ and *PKD2*^Δ17^ compared to their respective unedited (UE) parental ADPKD cultures. **g**, **h** Images showing higher mitotracker labeling (red) and reduced pCREB1 immunostaining (green) in *PKD1*^Δ17^ and *PKD2*^Δ17^ ADPKD cultures compared to their respective unedited parental ADPKD cultures. *n* = *3* biologically independent experiments for each cell line. Errors bars represent SEM, Statistical analysis: Two-tailed Students *t*-test (**e**, **f**). Source data are provided as a Source Data file.

A logical but crucial question is whether *PKD1/2* cis-inhibition is a feature of human ADPKD. We derived these cells from cysts of freshly discarded ADPKD nephrectomy samples from four affected individuals (three males aged 41, 48, and 52 years and one 57-year-old female). We performed *PKD1* and *PKD2* mutation analysis using DNA from cyst cells (Supplementary Fig. 16). Cell lines #1, #3, and #4 harbor heterozygous *PKD1* mutations, whereas cell line #2 harbors heterozygous truncating *PKD2* mutation and a missense heterozygous *PKD1* mutation. To assess the translational potential of our findings in mice, we used CRISPR/Cas9 editing to eliminate the *PKD1* or *PKD2* miR-17 motif in these primary ADPKD cultures (Supplementary Fig. 17). We designed human-specific sgRNAs targeting the miR-17 motifs in the *PKD1* or *PKD2* 3′-UTRs. We then transfected primary ADPKD cultures from all four donors with Cas9 and either *PKD1* or *PKD2* 3′-UTRs sgRNAs. Mock-transfected cells from each donor served as unedited parental controls. We noted higher PC1 levels within three days of modeling the *PKD1*^∆17^ allele in all four CRISPR-transfected cultures compared to their respective mock-transfected parental controls (Fig. 6a). Similarly, modeling *PKD2*^∆17^ alleles led to higher PC2 expression in CRISPR-transfected cultures than their respective mock-transfected parental controls (Fig. 6b). We next assessed the functional significance of *PKD1* or *PKD2* derepression by performing Matrigel 3D cystogenesis, alamarBlue proliferation assays, live-cell MitoTracker labeling, and anti-pCREB1 immunofluorescence. The CRISPR-transfected cultures containing *PKD1*^∆17^ or *PKD2*^∆17^ cells formed smaller cysts (Fig. 6c–f) and exhibited lower proliferation rates (Supplementary Fig. 18), higher MitoTracker signal, and lower pCREB1 expression compared to their respective mock-transfected, unedited controls (Fig. 6g, h). These data imply ongoing *PKD1/2* cis-inhibition and demonstrate the potential benefit of derepressing *PKD1/2* in human ADPKD cells

## Discussion

Heterozygous *PKD1* loss-of-function as the genetic cause of ADPKD was discovered over 25 years ago^31–33^, but approaches to restore *PKD1* expression have remained elusive. In this work, we provide a feasible framework for increasing endogenous *PKD1* levels and show for the first time that monoallelic *Pkd1* derepression is sufficient to alleviate preclinical PKD.

A unifying and parsimonious explanation for ADPKD onset is that cystogenesis ensues when the functional *PKD1* dosage falls by 70–80%, dipping below a critical threshold^3^. Thus, germline inactivation of one *PKD1* allele alone cannot account for this magnitude of dose reduction. Additional stochastic events that repress the remaining allele are required and play a critical role in determining disease onset. In this regard, we discovered that miR-17-mediated inefficient translation of mRNAs transcribed by the non-inactivated *PKD1* allele represents a targetable, somatic inhibitory ADPKD onset mechanism. As an attractive safety feature, *Pkd1* inhibition by miR-17 appears to be an ADPKD-specific phenomenon since we observed that the miR-17 level is low in normal adult mouse kidneys, and thus, it has no impact on *Pkd1* mRNA stability in the non-cystic setting. In contrast, the miR-17 miRNA family becomes activated in PKD models, where it appears to mediate *Pkd1* repression well into adulthood, as evidenced by the attenuation of cyst growth by the anti-miR-17 drug RGLS4326, even if the treatment is initiated at later stages of the disease. A noteworthy caveat here is that while RGLS4326 raises PC1 levels, its benefits in later stages of disease could be derived from simultaneous derepression of other miR-17 targets, including PC2 and Ppara. Another insight from our work is that potentially restoring hypomorphic *Pkd1* mutants may be a beneficial therapeutic approach. On a cautionary note, particularly for modalities employing exogenous *PKD1* supplementation, raising *Pkd1* above wildtype levels produces cystic disease in mice^34,35^. However, the uniqueness of our method is that, rather than transactivation, it relies on preventing inhibition, making it unlikely that *PKD1* will rise to the supratherapeutic range. As a sign that the miR-17-mediated *PKD1* inhibition may even be relevant in individuals with ADPKD, we noted that deleting the *PKD1* miR-17 motif in primary human ADPKD cultures increases PC1, and reduces 3D cyst growth and proliferation. Similarly, inhibiting miR-17 raises PC1 levels and inhibits the cyst growth and proliferation of primary human ADPKD cultures^4,30^. As further proof, a recent phase 1b clinical trial showed that RGLS4326 treatment is associated with higher urinary PC1 levels in ADPKD patients^36^. Additional clinical studies are planned using the next-generation anti-miR-17 oligo RGLS8429.

Our studies also clarify the role of *PKD1* in the continual expansion and growth of kidney cysts. Along with cyst initiation, *PKD1* inhibition unleashes large-scale transcriptomic and metabolic dysregulation and activates numerous oncogenic pathways, such as cAMP and c-Myc/Yap^4,37–45^. In turn, this downstream cyst-pathogenic signaling is thought to fuel cyst expansion. Despite such widespread dysregulation, a recent elegant study reported that transgenic *Pkd1* or *Pkd2* reconstitution rapidly reverts established cystic disease in mice^46^. Consistently, we found that acute *Pkd1/2* derepression reigns in established cystic disease and makes *Pkd1*-mutant cells resistant to pro-cystogenic stimuli such as cAMP and SAM. These observations collectively point to *PKD1* as the primary, if not the sole, factor governing cyst onset and growth.

We report an unexpected finding that *Pkd2* influences the cystic phenotype of *Pkd1*-mutant models. PC1 and PC2 physically interact and are coexpressed at multiple subcellular locations, indicating that the two proteins function in the same physiological pathway^47–50^. We add a new dimension by extending this relationship into the pathological context. Perhaps, enhancing *Pkd2* expression in *Pkd1*-mutant cells may improve PC1 trafficking and/or form more heteromeric PC1-PC2 protein complexes.

Finally, our work provides new insights into miRNA biology. miRNAs are well known to simultaneously but subtly repress large mRNA networks. Our approach decouples and disentangles this pleiotropy in the context of PKD. We devised a system where miR-17 is prevented from binding to *Pkd1* (or *Pkd2*) while its ability to interact with its other mRNA targets remains intact. Strikingly, eliminating just one 3′-UTR miR-17 motif phenocopies the effects of inhibiting all of miR-17 in *Pkd1*^*RC/*-^ models. Thus, our work is among the first to show that, in some circumstances, the majority of the biological effect of a miRNA can be derived through the repression of a handful of its targets. There are some parallels between our work and the miR-122 - Hepatitis C (HCV) infection axis in terms of targeting the disease-central RNA. However, the miR-122 mechanism is unconventional because it targets the foreign HCV RNA genome, binds the 5′-UTR, and aids in HCV accumulation^51,52^.

Most miRNAs are dispensable for homeostatic tissue functions and are pharmaceutically inhibited with relative ease. Despite these favorable characteristics, miRNA-based drug development has languished compared to other forms of RNA therapeutics^53^. This is partly because the pleiotropic molecular mechanism of numerous downstream mRNA targets makes it difficult to validate the miRNA biological effect or develop pharmacodynamic readouts of anti-miRNA drugs. We argue that prioritizing miRNAs that function as tonic inhibitors of a handful of disease-central mRNAs is likely to be a fruitful drug development strategy. Importantly, our insights are transferable, and we speculate that similar modes of therapeutically targetable cis-inhibitory regulation exist in other disorders, especially haploinsufficient monogenetic conditions.

## Methods

### Generation of 3′-UTR cell lines via CRISPR/Cas9

We deleted the miR-17 binding site from the *Pkd1* or *Pkd2* 3′-UTR using CRISPR/Cas9. We designed sgRNAs using www.benchling.com and ordered the sequences from IDT. The sgRNA pair targeted DNA sequences upstream and downstream of the miR-17 motif in the *Pkd1* or *Pkd2* genes. We cloned the sgRNAs into the CRISPR mammalian expression vector pSpCas9(BB)−2A-GFP^54^. Using these sgRNA encoding plasmids, we generated the *Pkd1*^*RC*∆17/-^ cell line and the *Pkd1*^*RC*/-^; *Pkd2*^∆17/∆17^ cell line as described next. To generate the *Pkd1*^*RC*∆17/-^ cell line, we transfected *Pkd1*^RC/-^ cells with 0.6 µg of the SpCas9-2A-GFP plasmid carrying the upstream or the downstream sgRNA using Lipofectamine 3000. After 72 h, we performed FACS to select GFP-positive cells with the top 5% intensity. These cells underwent clonal expansion in 96-well plates. Well-formed colonies were screened for the absence of the miR-17 binding site by DNA PCR of the targeted *Pkd1* genomic sequence. Clones with expected deletion bands were confirmed by Sanger sequencing. Two *Pkd1*^*RC*∆17/-^ clonal cell lines with confirmed deletions were further characterized and analyzed along with their parental control cell lines, as shown in Fig. 2 and Supplementary Fig. 4. We used the same strategy and experimental approach for generating the two *Pkd1*^*RC*/-^; *Pkd2*^∆17/∆17^ cell lines (Fig. 4 and Supplementary Fig. 8). The sgRNA sequences and genotyping primers are provided in Supplementary Table 3.

### Generation of 3′-UTR mice via CRISPR/Cas9

The following strains of mice were used: (1) for the mouse models shown in Fig. 1, wildtype C57BL/6 N female and male mice were used; (2) for the mouse models shown in Figs. 2 and 4, we used *Ksp*^Cre^; *Pkd1*^RC/RC^ mice maintained on a C57BL/6 J background by our laboratory. Prepubertal female mice underwent superovulation using a standard hormone regimen. The epididymis was collected from male mice for sperm harvest. After in vitro fertilization, one-cell fertilized eggs were isolated. CRISPR reagents (IDT) were delivered to the cytoplasm via electroporation using a Nepa21 Super Electroporator (NEPAGENE, Ichikawa, Japan). The eggs that survived the electroporation were washed and cultured in fresh M16 media in microdrop cultures. The eggs were then surgically transferred into the oviducts of day 1 pseudopregnant ICR females. At 21 days of age, founder mice were screened for deletion of the miR-17 binding site by genotyping, and confirmation of deletion was performed by Sanger sequencing.

### ADPKD mouse models

*Ksp*^Cre^, *Pkd1*^F/F^, and *Pkd1*^RC/RC^ mice were used in this study^4,24,30,55^. All mice were maintained on a C57BL/6 J background. At prespecified time points, mice were anesthetized using an approved protocol, and blood was obtained via cardiac puncture. The right kidney was weighed to obtain the KW/BW ratio and immediately flash frozen for future molecular analysis. The left kidney was perfused with ice-cold 1X PBS and 4% (wt/vol) paraformaldehyde. The kidney was subsequently paraffin-embedded. All studies used equal numbers of males and females. The UT Southwestern Institutional Animal Care and Use Committee approved all experiments involving animals.

### Pkd1^RC/+^ and Pkd1^RC/-^ cell lines

The *Pkd1*^*RC/+*^ and *Pkd1*^*RC/-*^ are isogenic, collecting duct-derived epithelial cell lines. These cells were generated from the kidneys of a 14-day-old *Pkd1*^RC/flox^ male mouse. A single-cell suspension was created by mincing the kidney tissue into 1 mm cubes followed by incubation for 40 min in DMEM containing 5% Collagenase (Sigma #C1639, USA) at 37 °C with intermittent agitation. The cells were then incubated with Biotinylated Dolichos Biflorus Agglutinin (DBA, a collecting duct marker) (Vector labs #B-1035) for 1 h. DBA-positive cells were isolated using a CELLection Biotin binder kit (Invitrogen #11533D). Subsequently, the cells were immortalized using the SV40 T Antigen Cell Immortalization Kit (Alstem #CILV01). One SV40-positive, immortalized *Pkd1*^*RC/Flox*^ clone was infected with an adenovirus that expresses Cre recombinase (Vector Biolabs #1779) to delete the floxed allele, thereby generating the *Pkd1*^*RC/-*^ cells. Recombination of the floxed allele was confirmed by genotyping. The uninfected parental clone with the genotype *Pkd1*^*RC/+*^ (where the ‘+‘ is the floxed allele) serves as the control. These cells are maintained in an epithelial culture medium (Dulbecco’s modified Eagle’s medium/Ham’s F-12 medium supplemented with 2% fetal bovine serum, insulin (8.3 × 10-7 m), prostaglandin E1 (7.1 × 10-8 m), selenium (6.8 × 10-9 m), transferrin (6.2 × 10-8 m), triiodothyronine (2 × 10-9 m), dexamethasone (5.09 × 10-8 m), and recombinant γ-interferon (10 units/ml) at 37 °C.

### Generation of the *Pkd1*^+/+^ and *Pkd1*^-/-^ cell lines

*Pkd1*^+/+^ and *Pkd1*^-/-^ cells are isogenic, renal tubule-derived epithelial cell lines. These cells were generated from the kidneys of a 12-day-old *Pkd1*^F/F^ male mouse pup. Kidneys were isolated and minced into 1 mm cubes. The tissue was incubated for 40 min in DMEM containing 5% Collagenase (Sigma #C1639, USA) at 37 °C with intermittent agitation to create a single-cell suspension. The cells were then strained using a 40-micron cell strainer and incubated with Biotinylated Dolichos Biflorus Agglutinin (DBA) (Vector labs #B-1035) for 1 h. DBA-positive cells were isolated using a CELLection Biotin binder kit (Invitrogen #11533D). Subsequently, the cells were immortalized using the SV40 T Antigen Cell Immortalization Kit (Alstem #CILV01) and cultured through clonal expansion. Clones were screened for SV40 marker by genotyping for SV40, and one clone was selected for further culture. *Pkd1*^-/-^ cells were generated by infecting the *Pkd1*^F/F^ cells with an adenovirus that expresses Cre recombinase (Vector Biolabs #1779) such that the floxed alleles are ablated. Infected cells were cultured through clonal expansion. The clones were genotyped to confirm successful recombination and deletion of both *Pkd1* alleles. The parent *Pkd1*^F/F^ and the *Pkd1*^-/-^ cells were further characterized through qRT-PCR and western blot analysis (Supplementary Fig. 2). These cells are grown and maintained in an epithelial cell culture medium as described in the section above.

### Histology

Tissue embedding in paraffin and subsequent sectioning were performed using standard protocols by the Histology core at UT Southwestern Medical Center. The tissues were cut into 5 µm sections and stained with hematoxylin-eosin (H&E) for histological analysis. The stained sections were imaged using a slide scanner.

### RNA

A Qiagen miRNEASY kit was used for total RNA extraction. cDNA was prepared using an Invitrogen First Strand Superscript III cDNA synthesis kit. Q-PCR was performed using iQ SYBR Green Supermix (Bio-Rad). All samples were loaded in duplicate or triplicate on the CFX ConnectTM Real-time PCR detection system. 18s was used to normalize mRNA expression. The sequences of the primers are shown in Supplementary Table 4.

### PC1 and other Western blots

Total protein was isolated from kidneys or cells using a lysis buffer made by mixing T-PER tissue protein extraction reagent (Invitrogen, catalog# 78510) with a protease phosphatase inhibitor tablet (Fisher, catalog# PIA32961) according to the manufacturer’s instructions. The lysis buffer was prepared and stored as one-time aliquots at −80 °C. The aliquots were thawed on ice immediately before protein isolation. Protein concentration was measured using the Bradford Assay reagent. Protein samples were prepared in 4X NuPAGE LDS Sample Buffer with 0.5% b-mercaptoethanol (Sigma, catalog# M6250) for all proteins except for PC1 and PC2 and their loading control beta-actin, which were prepared with 0.1 M DTT (Sigma, catalog# D0632). The samples were always freshly prepared before gel electrophoresis. BME samples were boiled for 5 min at 98 °C before loading on gels. The DTT samples were incubated at 25 °C for 10 min before loading on gels.

For full-length PC1 detection, the samples were run on the NuPAGE™ 3–8% Tris-Acetate Protein Gel (Invitrogen, EA03785) at 160 V for 1.5 h on ice. A high molecular weight protein ladder (Invitrogen, catalog# LC5699) was used in each gel to track 460 kDa proteins. Electrophoretically separated proteins were transferred using the Invitrogen transfer system at 200 mAmps for 100 min on ice or at 4 °C. The samples containing 10 µg of protein were run on mini-PROTEAN SDS-polyacrylamide precast gels to detect other proteins. A standard molecular weight ladder was used in each gel to track protein sizes. The gels were run at 150 V until the dye ran out. The proteins were transferred to a nitrocellulose membrane using the Trans-Blot Semi-Dry Transfer system on the mixed MW program.

After completing the transfer, the membranes were blocked with 5% fat-free milk and probed overnight at 4 °C with primary antibodies. The membranes were washed three times with 1x TBS-Tween the next morning before and after probing for one hour with a secondary antibody. Goat-anti-rabbit or anti-mouse HRP-conjugated IgG was used as the secondary antibody. HRP-conjugated actin antibody (Sigma, catalog# a3854) was used to measure total protein. The blots were developed using the chemiluminescence substrate SuperSignal West Dura, ECL, or Femto from Pierce. The blots were developed using the Bio-Rad digital imager. The protein bands were quantified using Imagelab software from Bio-Rad. Each Western blot was repeated at least three times. Ten micrograms of protein from cells or kidneys were run on gels to detect <150 kDa proteins. 40–60 µg of protein was run on gels to detect heavy molecular weight (462 kDa) full-length PC1 protein. All the primary antibodies were used at a 1:1000 dilution, except for PC1 (used at 1:500), and the secondary antibodies were used at a 1:5000 dilution. The following primary antibodies were used: PC1 (7E12 Santa Cruz, catalog# sc-130554); PC1 E8-8C3C10 (Baltimore PKD core center), PC2 (gift from the Baltimore PKD Core); pCREB (Cell Signaling, catalog# 9198); c-Myc (Abcam, catalog# ab185656), YAP1 (Cell Signaling, catalog# 4912); Mettl3 (Invitrogen, catalog# MA5-27527).

### Immunofluorescence on tissue samples

Paraffin sections of kidney tissues were used for immunofluorescence staining. Briefly, the slides were deparaffinized by first baking at 60 °C for 1 h and then washing in Histo-clear (Fisher, HS-2001) three times for 5 min each. Next, the slides were re-hydrated through 100%, 95%, and 70% ethanol washes before incubation in 1X PBS. The slides were then subjected to antigen retrieval with sodium citrate. The slides were treated with sodium borohydride to quench autofluorescence for 40 min. The slides were washed in 1X PBS three times and then blocked in 1X PBS + 10% goat serum+0.1% BSA (antibody block) for at least 1-2 h at RT. Sections were incubated with primary antibodies overnight. Primary antibodies were diluted with antibody block at a 1:500 dilution. Slides were washed in 1X PBS three times for 5 min each, treated with Alexa Fluor secondary antibodies (diluted using the antibody block to a 1:500 dilution) for 1 h, and then washed three times for 5 min each. The slides were mounted using Vecta Shield containing Dapi. The slides were imaged using the Zeiss Compound Light microscope or the Zeiss Axioscan Z1 slide scanner. The following antibodies were used: DBA (Vector Labs, catalog# B-1035), THP (Biomedical Technologies, catalog# BT-590), LTA (Vector Labs, catalog# B-1325), MRC1 (Abcam, catalog# ab64693), pCREB1 (Cell Signaling, catalog# 9198), and pHH3 (Sigma, catalog# H0412). Processing, immunostaining, and imaging of slides were performed simultaneously within each experiment.

### Immunofluorescence on cells

Immunofluorescence staining was performed on cells grown on 8-chambered slides (Fisher, catalog# 154534PK). The cells were fixed with 100% ice-cold methanol for 5 min at 4 °C. The slides were washed with 1X PBS 3 times for 5 min. The cells were then blocked in 1X PBS + 10% goat serum+0.1% BSA + 0.1 M glycine+0.1% Tween 20 (antibody block) for at least 30 min at room temperature. Primary antibodies were diluted with antibody block at a 1:100 dilution and added to the slides for 2 h. Slides were washed in 1X PBS three times for 5 min each, treated with Alexa Fluor secondary antibodies (diluted using the antibody block to a 1:500 dilution) for 1 h, and then washed three times for 5 min each. The slides were counterstained in DAPI (Fisher, catalog# ICN15757410) diluted at 1:10000 in distilled water for 10 min before imaging under a Zeiss Compound Light microscope. For each experiment, the control and treatment cells or the control and the ∆17 cells were seeded simultaneously on different chambers of the same slide. Processing, immunostaining, and imaging of slides were also performed simultaneously.

### MitoTracker analysis

MitoTracker Red CMXRos (Thermo Fisher, catalog# M7512) was used to analyze the mitochondrial membrane potential in live cells. The lyophilized MitoTracker® product was dissolved in dimethylsulfoxide (DMSO) to a final concentration of 1 mM and stored at −20 °C in small aliquots. Cells grown to 40–70% confluency were washed with sterile PBS and then treated with regular DMEM serum-free media containing 100 nM MitoTracker for 8 min. Immediately thereafter, the media was replaced with regular growth media and imaged under a Zeiss Compound Light microscope. The images were taken at the same exposure time for the samples of the same experiment. The intensity of fluorescence is directly proportional to membrane potential.

### 3D cystogenesis assay

25 µl of 100% Matrigel (Fisher, catalog# 354234) was spread onto each well of an 8-chambered slide with precooled 200 µl sterile pipette tips. The plate was then placed in a 37 °C incubator for 30 min for the Matrigel to set. In the interim, cells were trypsinized, washed once with PBS, filtered through a 40 µm cell strainer to create a single-cell suspension, and counted. Cells were seeded on the Matrigel-coated slide at a seeding density of 5000 cells/well in a 300 µl volume of growth media containing 2% Matrigel. For each cell line or treatment condition, cells were seeded in triplicate and incubated at 37 °C for 7 days to allow for the growth of 3D cysts in suspension. During this time, the wells were supplemented with growth medium 72 h after initial placement into Matrigel. On day 7, the chamber slides were imaged on a Leica DMI 3000B light microscope. The images were analyzed using ImageJ software to obtain cyst size measurements. Each assay was repeated at least three times. Measurements from each experiment were combined and analyzed for statistical significance.

### Ex vivo organ culture

Female mice bearing potential *Pkd1*^**+/+**^; *Pkd1*^**∆17/+**^, and *Pkd1*^**∆17/∆17**^ embryos were dissected at embryonic day (E) 13.5 in PBS to harvest the kidneys and tail. The tail of each embryo was used for DNA extraction and subsequent genotyping. The kidneys were set up for culture on Whatman membranes (Sigma, catalog# WHA110409) in an air-medium interface as described^28^. The kidneys were cultured in basal DMEM (Thermo Fisher, catalog# 12500) containing 10% fetal bovine serum (FBS), 2% PenStrep (Invitrogen, catalog# 1514022), 5 μg/ml insulin, 5 μg/ml transferrin, 2.8 nM sodium selenite, 25 ng/ml prostaglandin E and 32 pg/ml T3. One kidney was grown in the above media, and the contralateral kidney was grown in 100 µM 8-Br-cAMP (Sigma, catalog# B7880)-supplemented media. Using a second cohort of mice, one kidney was grown in 100 µM 8-Br-c-AMP or 100 µM 8-Br-c-AMP + 250 µM SAM. For all the cultures, the media was changed every 48 h. The cultures were imaged live using the Zeiss Stereo Lumar microscope on day 4. The cysts were measured and analyzed using ImageJ software. At the end of 6 days, the kidneys were flash-frozen and stored at −80 °C until further use for RNA or protein extraction.

### alamarBlue assay

*Pkd1*^**RC/-**^ and *Pkd1*^**RC∆17/-**^ cells (3 ×10^3 density) were seeded on 96-well plates. The next morning, the medium was changed to contain 1X alamarBlue reagent (Invitrogen, catalog# DAL1025) and vehicle, 100 µM 8-Br-cAMP, 100 µM SAM, or 17 mM glucose. Colorimetric readings were taken at 570 nm and 600 nm in a microplate reader after 12 h. The redox reaction of alamarBlue was used to assess cell proliferation quantitatively. N = 8 was used for each condition. The values were plotted as a scaled heatmap using the Python MatplotLib package. The same experimental approach was used for the *Pkd1*^**RC∆/-**^; *Pkd2*^**∆17/∆17**^ cells and the control cells *Pkd1*^**RC/-**^; *Pkd2*^**+/+**^.

### Serum electrolytes

Serum creatinine was measured by capillary electrophoresis by the UT Southwestern O’Brien Center. BUN was measured by Vitros250 Analyzer by the UT Southwestern Metabolic Phenotyping Core.

### Microarray analysis of microRNAs

Total RNA was extracted from kidneys using miRNeasy mini kits (Qiagen). The small RNA fraction (<300 nucleotides) was hybridized on a μParaflo Microfluidic chip containing detection probes for all mouse microRNAs (miRNAs) in the miRBase version-17 (miRBase, http://microrna.sanger.ac.uk/sequences). The hybridized microarray chips were labeled with fluorescent dyes and laser scanned to obtain fluorescent images. The signal values for each sample were derived by background subtraction and normalization. Microarray chip hybridization, fluorescent labeling, laser scanning, and background subtraction and normalization were performed by LC Sciences. The signal values from each of the five age groups (P2, P7, P14, P35) were averaged, and the P values using One-way ANOVA were calculated. The differentially detected signals are defined by *P* < 0.05.

### RNA-seq preprocessing

Sequencing quality control was performed with FastQC v0.11.8. RNA-seq reads were trimmed, and low-quality reads were removed using Trimgalore v0.6.3_dev (https://www.bioinformatics.babraham.ac.uk/projects/trim_galore/) with the “paired” parameter and length of 150 bps. Trimmed fastq sequences were aligned to the mouse reference genome GRCm38 using STAR aligner v2.5.3a with the produced bam files sorted by coordinate by using the option “--outSAMtype BAM SortedByCoordinate. ^56,57^“ Raw read gene counts were obtained using STAR aligner with the options “—quantMode GeneCounts” and “--sjdbGTFfile” with gene models in GTF format obtained from mouse EnsEMBL release 94. Alignment quality control and read mapping statistics were obtained from Picard tools v2.20.3 using the function “CollectMultipleMetrics” (http://broadinstitute.github.io/picard/).

### RNA-seq data analysis

Raw gene counts were used for quality control and differential expression analysis. Raw counts were normalized to the total number of reads by calculating log_2_CPM (counts per million). We carefully examined the log_2_CPM distribution and its relationship to the standard deviation and determined the appropriate cutoff (average Log_2_CPM < −3) to eliminate lowly expressed genes before differential gene expression analysis. TPM (transcript per million) quantification was performed using RSEM v1.3.1, and differential gene expression analysis was performed using the limma-trend (version 3.40.6) in R^58,59^.

### Transcript quantification

Individual *Pkd1* transcript quantification was performed using Salmon v1.3.0^60^. The five different transcript versions for *Pkd1* were added to the RefSeq mm9 fasta reference transcriptome to build a novel Salmon index. Then, the fastq files were directly mapped and read counts, and TPM values were quantified with the standard process.

### In vitro RGLS4326 experiments

*Pkd1*^**RC/-**^ cells were seeded at 2 ×10^5 confluence in 6-well plates. The next morning, cells were transfected using Lipofectamine 3000 with a vehicle, control oligonucleotide, or RGLS4326 at a final concentration of 100 µM. Forty-eight hours after transfection, the cells were collected for RNA extraction. Seventy-two hours after transfection, cells were harvested for protein or further seeded for alamarBlue assay and 3D cystogenesis assay. For the experiment shown in Fig. 5C, the 3D cystogenesis assay was performed with untreated *Pkd1*^**RC/-**^ cells, as described in the methods section of the 3D cystogenesis assay with the following changes. On day 4 of Matrigel culture, the wells were imaged using the Leica light microscope DMI 3000B, and then the cultures were transfected with a vehicle, 100 µM control oligonucleotide, or 100 µM RGLS4326 and grown for 3 additional days. On day 7, the samples underwent imaging to assess cyst size.

### RGLS4326 mouse experiments

The Ksp^Cre^; *Pkd1*^F/RC^ mouse line was used for the drug studies. Mice were randomly assigned and administered 20 mg kg^−1^ vehicle (PBS), control oligonucleotide, or RGLS4326 via subcutaneous injections. For the first cyst prevention study (Fig. 5D–G), mice were injected on postnatal days (P) 10, P11, P12, and P16 and sacrificed on P18. Nontransgenic strain-matched mice were also sacrificed on the same days. For the second disease stabilization study (Fig. 5H–K), mice were injected at P16 and P17 and sacrificed at P26. One mouse from the study succumbed to the disease and died earlier than 26 days of age. For the third long-term study (Fig. 5L–N), mice were injected on P16 and P17 and then every week until 18 weeks of age. Another cohort of mice received the same dose of RGLS4326 treatment on P16 and P17 and then semimonthly thereafter until 18 weeks of age. The mice were observed every day for 18 weeks to note death. At the end of 18 weeks, the surviving mice were sacrificed to harvest tissue. Equal numbers of males and females were used in all study groups.

### Human ADPKD cell experiments

Primary human ADPKD cyst cells were obtained from PKD Research Biomarker and Biomaterial Core at the University of Kansas Medical Center (KUMC). The use of surgically discarded kidney tissues complied with federal regulations and was approved by the Institutional Review Board at the University of Kansas Medical Center. *PKD1* and *PKD2* mutation analysis of DNA from donor cyst cells was performed by Ambry Genetics (Aliso Viejo, CA). Each primary cell line was cultured in DMEM/F12 + + (Gibco, catalog# 10565–018) supplemented with 10% FBS, 5 μg kg^−1^ insulin, 5 μg mL^−1^ transferrin, and 5 ng mL^−1^ sodium selenite and incubated in an atmosphere of 95% air and 5% CO2 at 37 °C until 80% confluency. At the 2^nd^ passage, cells from each human donor underwent reverse transfection using CRISPRMAX reagent (Invitrogen) containing Cas9 protein (IDT) and synthetic sgRNAs (IDT) or were transfected with vehicle (lacking Cas9). The Cas9/sgRNA-transfected cultures are a mixed population of edited and unedited cells. Clonal propagation was not possible because these are primary cells that allow only a limited number of passages. Cas9- or vehicle-transfected cells (control) were then seeded into 6-well plates and chamber slides. After 72 h, the cells were harvested for genotyping, Western blot analysis, and immunofluorescence/MitoTracker staining. In addition, at 72 h posttransfection, cells were trypsinized and plated at 4000 cells/well density in 200 μl of media plus Matrigel (Corning, catalog# 354234) in a 96-well plate (Corning, catalog# 353072). Media was replenished 72 h after the initial placement into Matrigel. Cyst images were obtained on the 7^th^ day of Matrigel culture (10^th^ day after Cas9/SgRNA or vehicle transfection). One hundred cyst images were obtained for Cas9- or vehicle-transfected cells from each donor. Similarly, 72 h post-transfection, cells were seeded at 2000 cells/well density in 96-well plates for the alamarBlue proliferation assay. The following day, the media was replaced with growth media containing 1X alamarBlue, and readings were taken 12 h later.

### Statistics and reproducibility

All experiments were carried out with at least three biological replicates and showed successful reproducibility. For in vivo experiments, *N* is the number of mice analyzed. For in vitro experiments, *N* refers to the number of biological replicates. Two-tailed Student’s *t*-test was used for pairwise comparisons and analysis of variance (ANOVA), followed by Tukey’s post hoc test was used for multiple comparisons. The Mantel-Cox test was used for the analysis of mouse survival. All data were analyzed using Prism software (GraphPad Software). *P* < 0.05 was considered statistically significant. The sample size and *P* values are mentioned in the figure graphs, the figure legends, or the results section. For the RGLS4326 studies, animals were randomly assigned to treatment arms. Investigators were not blinded to the treatment or the genotypes of the animals.

### Reporting summary

Further information on research design is available in the Nature Research Reporting Summary linked to this article.

## Supplementary information

Supplementary Information

Reporting Summary

## Data Availability

The data that support this study are available from the corresponding author upon request. The RNA-seq datasets have been deposited in the NCBI Gene Expression Omnibus repository under accession number GSE196237. The microarray dataset has been deposited in the NCBI Gene Expression Omnibus repository under accession number GSE208429. Source data are provided with this paper.

## Source data

Source data

## References

[1] Patel V, Chowdhury R, Igarashi P (2009). Advances in the pathogenesis and treatment of polycystic kidney disease. Curr. Opin. Nephrol. Hypertens..

[2] Lanktree MB, Haghighi A, di Bari I, Song X, Pei Y (2021). Insights into Autosomal Dominant Polycystic Kidney Disease from Genetic Studies. Clin. J. Am. Soc. Nephrol..

[3] Ong AC, Harris PC (2015). A polycystin-centric view of cyst formation and disease: the polycystins revisited. Kidney Int.

[4] Hajarnis S (2017). microRNA-17 family promotes polycystic kidney disease progression through modulation of mitochondrial metabolism. Nat. Commun..

[5] Ward CJ (1996). Polycystin, the polycystic kidney disease 1 protein, is expressed by epithelial cells in fetal, adult, and polycystic kidney. Proc. Natl Acad. Sci. USA.

[6] Ong AC (1999). Polycystin-1 expression in PKD1, early-onset PKD1, and TSC2/PKD1 cystic tissue. Kidney Int.

[7] Geng L (1996). Identification and localization of polycystin, the PKD1 gene product. J. Clin. Invest.

[8] Cornec-Le Gall E (2013). Type of PKD1 mutation influences renal outcome in ADPKD. J. Am. Soc. Nephrol..

[9] Rossetti S (2009). Incompletely penetrant PKD1 alleles suggest a role for gene dosage in cyst initiation in polycystic kidney disease. Kidney Int.

[10] Lanktree MB (2021). Patients with Protein-Truncating PKD1 Mutations and Mild ADPKD. Clin. J. Am. Soc. Nephrol..

[11] Hopp K (2012). Functional polycystin-1 dosage governs autosomal dominant polycystic kidney disease severity. J. Clin. Invest.

[12] Lantinga-van Leeuwen IS (2004). Lowering of Pkd1 expression is sufficient to cause polycystic kidney disease. Hum. Mol. Genet.

[13] Jiang ST (2006). Defining a link with autosomal-dominant polycystic kidney disease in mice with congenitally low expression of Pkd1. Am. J. Pathol..

[14] Wang E (2010). Progressive renal distortion by multiple cysts in transgenic mice expressing artificial microRNAs against Pkd1. J. Pathol..

[15] Tsukiyama T (2019). Monkeys mutant for PKD1 recapitulate human autosomal dominant polycystic kidney disease. Nat. Commun..

[16] Watanabe, M. et al. Generation of heterozygous PKD1 mutant pigs exhibiting early-onset renal cyst formation. *Lab Invest*, 10.1038/s41374-021-00717-z (2022). 10.1038/s41374-021-00717-z PMC9042704 34980882

[17] Mayr, C. What Are 3’ UTRs Doing? *Cold Spring Harb Perspect Biol* **11**, 10.1101/cshperspect.a034728 (2019). 10.1101/cshperspect.a034728 PMC6771366 30181377

[18] Baek D (2008). The impact of microRNAs on protein output. Nature.

[19] Bartel DP (2004). MicroRNAs: genomics, biogenesis, mechanism, and function. Cell.

[20] Bartel DP, Chen CZ (2004). Micromanagers of gene expression: the potentially widespread influence of metazoan microRNAs. Nat. Rev. Genet.

[21] Eisen TJ, Eichhorn SW, Subtelny AO, Bartel DP (2020). MicroRNAs Cause Accelerated Decay of Short-Tailed Target mRNAs. Mol. Cell.

[22] Agarwal, V., Bell, G. W., Nam, J. W. & Bartel, D. P. Predicting effective microRNA target sites in mammalian mRNAs. *Elife* **4**, 10.7554/eLife.05005 (2015). 10.7554/eLife.05005 PMC4532895 26267216

[23] Ramalingam H, Yheskel M, Patel V (2020). Modulation of polycystic kidney disease by non-coding RNAs. Cell Signal.

[24] Yheskel M, Lakhia R, Cobo-Stark P, Flaten A, Patel V (2019). Anti-microRNA screen uncovers miR-17 family within miR-17~92 cluster as the primary driver of kidney cyst growth. Sci. Rep..

[25] Patel V (2013). miR-17~92 miRNA cluster promotes kidney cyst growth in polycystic kidney disease. Proc. Natl Acad. Sci. USA.

[26] Yheskel M, Patel V (2017). Therapeutic microRNAs in polycystic kidney disease. Curr. Opin. Nephrol. Hypertens..

[27] Aguilar AL (2010). The small RNA expression profile of the developing murine urinary and reproductive systems. FEBS Lett..

[28] Ramalingam H (2021). A methionine-Mettl3-N(6)-methyladenosine axis promotes polycystic kidney disease. Cell Metab..

[29] Lakhia, R. et al. Enhancer and super-enhancer landscape in polycystic kidney disease. 10.1101/2021.11.19.469306 (2021). 10.1016/j.kint.2022.08.039 PMC9841439 36283570

[30] Lee EC (2019). Discovery and preclinical evaluation of anti-miR-17 oligonucleotide RGLS4326 for the treatment of polycystic kidney disease. Nat. Commun..

[31] Mochizuki T (1996). PKD2, a gene for polycystic kidney disease that encodes an integral membrane protein. Science.

[32] The polycystic kidney disease 1 gene encodes a 14 kb transcript and lies within a duplicated region on chromosome 16. (1994). The European Polycystic Kidney Disease Consortium. Cell.

[33] Hughes J (1995). The polycystic kidney disease 1 (PKD1) gene encodes a novel protein with multiple cell recognition domains. Nat. Genet.

[34] Thivierge C (2006). Overexpression of PKD1 causes polycystic kidney disease. Mol. Cell Biol..

[35] Patel V (2012). MicroRNAs regulate renal tubule maturation through modulation of Pkd1. J. Am. Soc. Nephrol..

[36] Lee E. et al RGLS4326 Increases Urinary PC1 and PC2 Levels in Individuals with Autosomal Dominant Polycystic Kidney Disease (ADPKD). *American Society of Nephrology Kidney Week* (2021).

[37] Lakhia R (2018). PPARalpha agonist fenofibrate enhances fatty acid beta-oxidation and attenuates polycystic kidney and liver disease in mice. Am. J. Physiol. Ren. Physiol..

[38] Kusminski CM (2012). MitoNEET-driven alterations in adipocyte mitochondrial activity reveal a crucial adaptive process that preserves insulin sensitivity in obesity. Nat. Med.

[39] Zimmerman KA (2019). Tissue-Resident Macrophages Promote Renal Cystic Disease. J. Am. Soc. Nephrol..

[40] Kleczko EK (2018). CD8(+) T cells modulate autosomal dominant polycystic kidney disease progression. Kidney Int.

[41] Karihaloo A (2011). Macrophages promote cyst growth in polycystic kidney disease. J. Am. Soc. Nephrol..

[42] Chen L (2015). Macrophage migration inhibitory factor promotes cyst growth in polycystic kidney disease. J. Clin. Invest.

[43] Ma S, Guan KL (2018). Polycystic kidney disease: a Hippo connection. Genes Dev..

[44] Happe H (2011). Altered Hippo signalling in polycystic kidney disease. J. Pathol..

[45] Wang Q (2018). Adenylyl cyclase 5 deficiency reduces renal cyclic AMP and cyst growth in an orthologous mouse model of polycystic kidney disease. Kidney Int.

[46] Dong K (2021). Renal plasticity revealed through reversal of polycystic kidney disease in mice. Nat. Genet.

[47] Su, Q. et al. Structure of the human PKD1-PKD2 complex. *Science* **361**, 10.1126/science.aat9819 (2018). 10.1126/science.aat9819 30093605

[48] Hanaoka K (2000). Co-assembly of polycystin-1 and −2 produces unique cation-permeable currents. Nature.

[49] Tsiokas L, Kim E, Arnould T, Sukhatme VP, Walz G (1997). Homo- and heterodimeric interactions between the gene products of PKD1 and PKD2. Proc. Natl Acad. Sci. USA.

[50] Qian F (1997). PKD1 interacts with PKD2 through a probable coiled-coil domain. Nat. Genet.

[51] Luna JM (2015). Hepatitis C virus RNA functionally sequesters miR-122. Cell.

[52] Mortimer SA, Doudna JA (2013). Unconventional miR-122 binding stabilizes the HCV genome by forming a trimolecular RNA structure. Nucleic Acids Res.

[53] Roberts TC, Langer R, Wood MJA (2020). Advances in oligonucleotide drug delivery. Nat. Rev. Drug Disco..

[54] Ran FA (2013). Genome engineering using the CRISPR-Cas9 system. Nat. Protoc..

[55] Lakhia, R. *et al*. Interstitial microRNA miR-214 attenuates inflammation and polycystic kidney disease progression. *JCI Insight* **5**, 10.1172/jci.insight.133785 (2020). 10.1172/jci.insight.133785 PMC7205276 32182218

[56] Ruffier, M. et al. Ensembl core software resources: storage and programmatic access for DNA sequence and genome annotation. *Database (Oxford)* **2017**, 10.1093/database/bax020 (2017). 10.1093/database/bax020 PMC5467575 28365736

[57] Dobin A (2013). STAR: ultrafast universal RNA-seq aligner. Bioinformatics.

[58] Li B, Dewey CN (2011). RSEM: accurate transcript quantification from RNA-Seq data with or without a reference genome. BMC Bioinforma..

[59] Ritchie ME (2015). limma powers differential expression analyses for RNA-sequencing and microarray studies. Nucleic acids Res..

[60] Patro R, Duggal G, Love MI, Irizarry RA, Kingsford C (2017). Salmon provides fast and bias-aware quantification of transcript expression. Nat. Methods.

