# Supplementary material for: PKD1 and PKD2 mRNA cis-inhibition drives polycystic kidney disease progression
Source: Nat Commun. 2022 Aug 15;13:4765. doi: 10.1038/s41467-022-32543-2 (PMC9376183; doi:10.1038/s41467-022-32543-2)
Supplement: Supplementary file 1 — Supplementary Information [file 41467_2022_32543_MOESM1_ESM.pdf]

SUPPLEMENTARY INFORMATION

PKD1 and *PKD2* mRNA cis-inhibition drives polycystic kidney disease progression

Ronak Lakhia[1]*, Harini Ramalingam[1]*, Chun-Mien Chang[1], Patricia Cobo-Stark[1], Laurence Biggers[1], Andrea Flaten[1], Jesus Alvarez[1], Tania Valencia[2], Darren P. Wallace[3], Edmund Lee[2], and Vishal Patel[1]

[1]Department of Internal Medicine, Nephrology, UT Southwestern Medical Center, Dallas, Texas, USA 75390.

[2]Regulus Therapeutics., San Diego, CA, 92121, USA.

[3]Department of Internal Medicine and the Jared Grantham Kidney Institute, University of Kansas Medical Center, Kansas City, Kansas, USA

* These authors contributed equally

Address correspondence: Vishal Patel, MD. Email: vishald.patel@utsouthwestern.edu

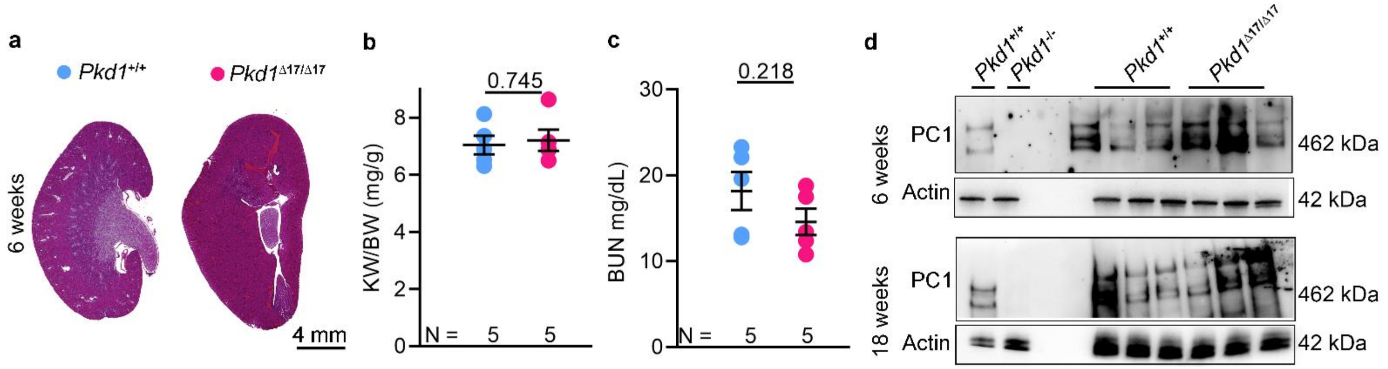

Supplementary Figure 1: Characterization of *Pkd1*$^{\Delta17/\Delta17}$ mice. a. H&E-stained kidney sections of 6-week-old *Pkd1*$^{+/+}$ and *Pkd1*$^{\Delta17/\Delta17}$ mice are shown (*n* = 5). **b & c.** Kidney-weight-to-body-weight (KW/BW) ratio and BUN levels of 6-week-old *Pkd1*$^{+/+}$ and *Pkd1*$^{\Delta17/\Delta17}$ mice are shown. **d.** Immunoblot showing PC1 expression in *Pkd1*$^{+/+}$, *Pkd1*$^{-/-}$ cell lines and in kidneys of 6 or 18-week-old *Pkd1*$^{+/+}$ and *Pkd1*$^{\Delta17/\Delta17}$ mice. (*n* = 5 for each genotype). Lysates from *Pkd1*$^{-/-}$ cells serve as negative control and do not exhibit PC1 expression. Error bars indicate SEM. Statistical analysis two-tailed Student's t-test (b-c).

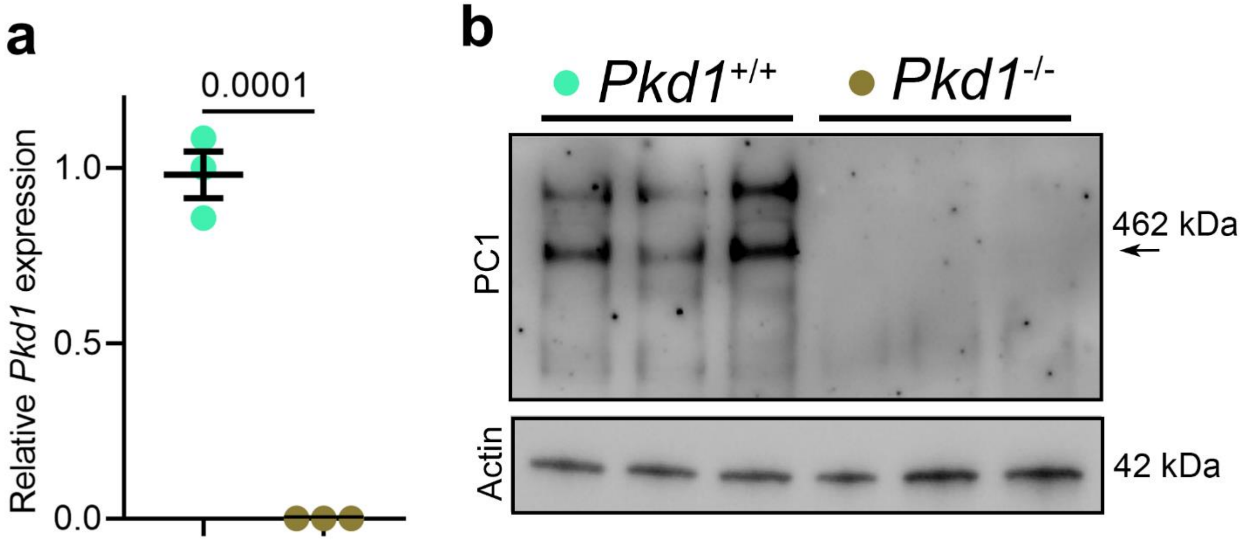

Supplementary Figure 2: Validation of the 7E12 PC1 antibody. We tested the 7E12 PC1 antibody in *Pkd1*$^{+/+}$ and *Pkd1*$^{-/-}$ collecting duct cell lines. See methods for details on the antibody and the cell lines. **a.** qRT-PCR showing that the *Pkd1*$^{-/-}$ cells do not express the *Pkd1* mRNA. **b.** Immunoblot showing absence of full-length PC1 protein in the *Pkd1*$^{-/-}$ cell line. n = 3 biologically independent samples from the indicated cell lines. Error bars indicate SEM. Statistical analysis two-tailed Student's t-test (a).

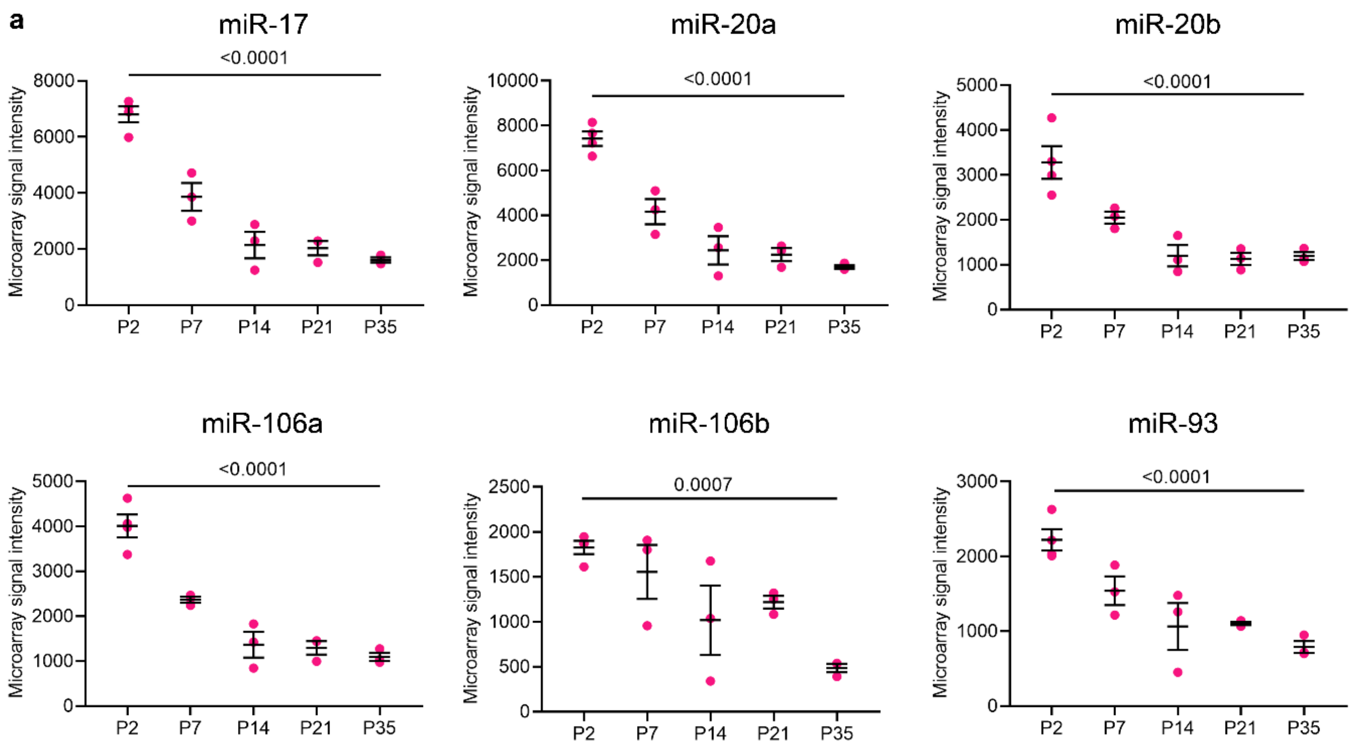

Supplementary Figure 3: miR-17 family expression declines with postnatal kidney maturation. a. Microarray signal intensity values for miRNAs belonging to the miR-17 family, miR-17, miR-20a, miR-20b, miR-106a, miR-106b, or miR-93 in mouse kidneys at ages P2, P7, P14, P21, and P35. The miR-17 family members show an age-dependent decrease in expression. n = 4 mouse kidneys at P2, n = 3 mouse kidneys at P7, P14, P21 or P35. Error bars indicate SEM. Statistical analysis one-way *ANOVA*, test for linear trend.

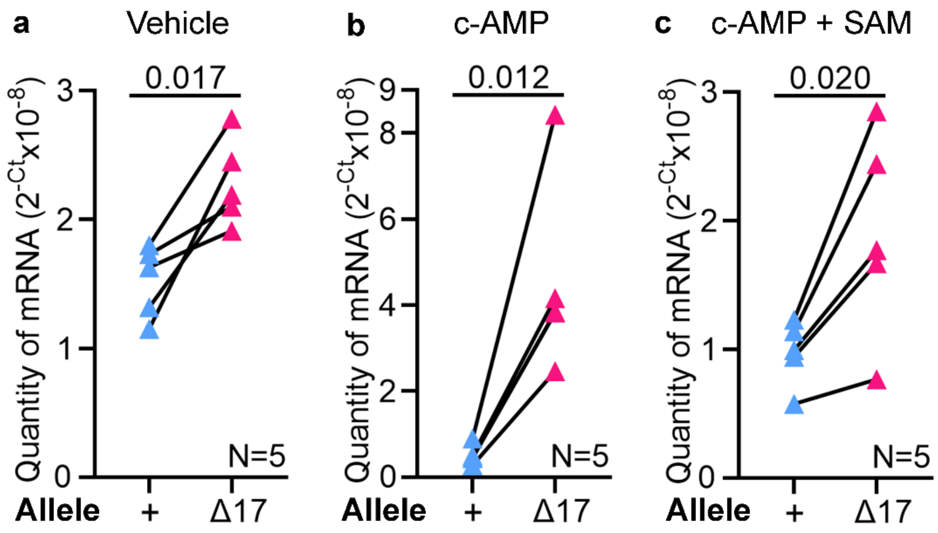

Supplementary Figure 4: *Pkd1* is cis-inhibited via its miR-17 3'-UTR motif. Allele-specific qRT-PCR analysis showing the quantity of *Pkd1* mRNAs produced by the wildtype (+) and Δ17 alleles in *ex vivo* kidney cultures of *Pkd1*$^{\Delta 17/+}$ mice treated with vehicle (**a**), c-AMP (**b**), or c-AMP plus SAM (**c**). The *Pkd1* $^{\Delta 17}$ allele produced more mRNA transcripts compared to the *Pkd1*$^{+}$ allele. This difference was even more pronounced in the presence of c-AMP. *n* = 5 *ex vivo* kidney cultures. Statistical analysis: paired t-test. Error bars indicate SEM.

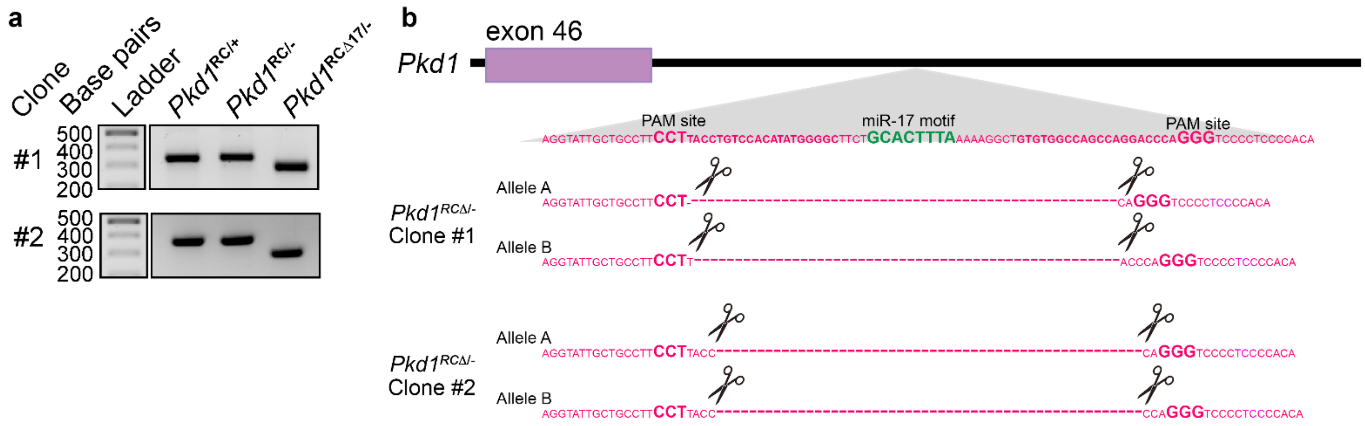

Supplementary Figure 5: Characterization of CRISPR-edited *Pkd1*$^{RC/-}$ cell lines. **a.** PCR products obtained after amplifying the DNA (encoding the *Pkd1* 3'UTR segment) from parental and CRISPR-edited cell lines. The lower band indicates the $\Delta 17$ genotype. **b.** Graphical illustration of Sanger sequencing results from the $\Delta 17$ bands of each clone confirm deletion of the miR-17 motif from both *Pkd1* alleles.

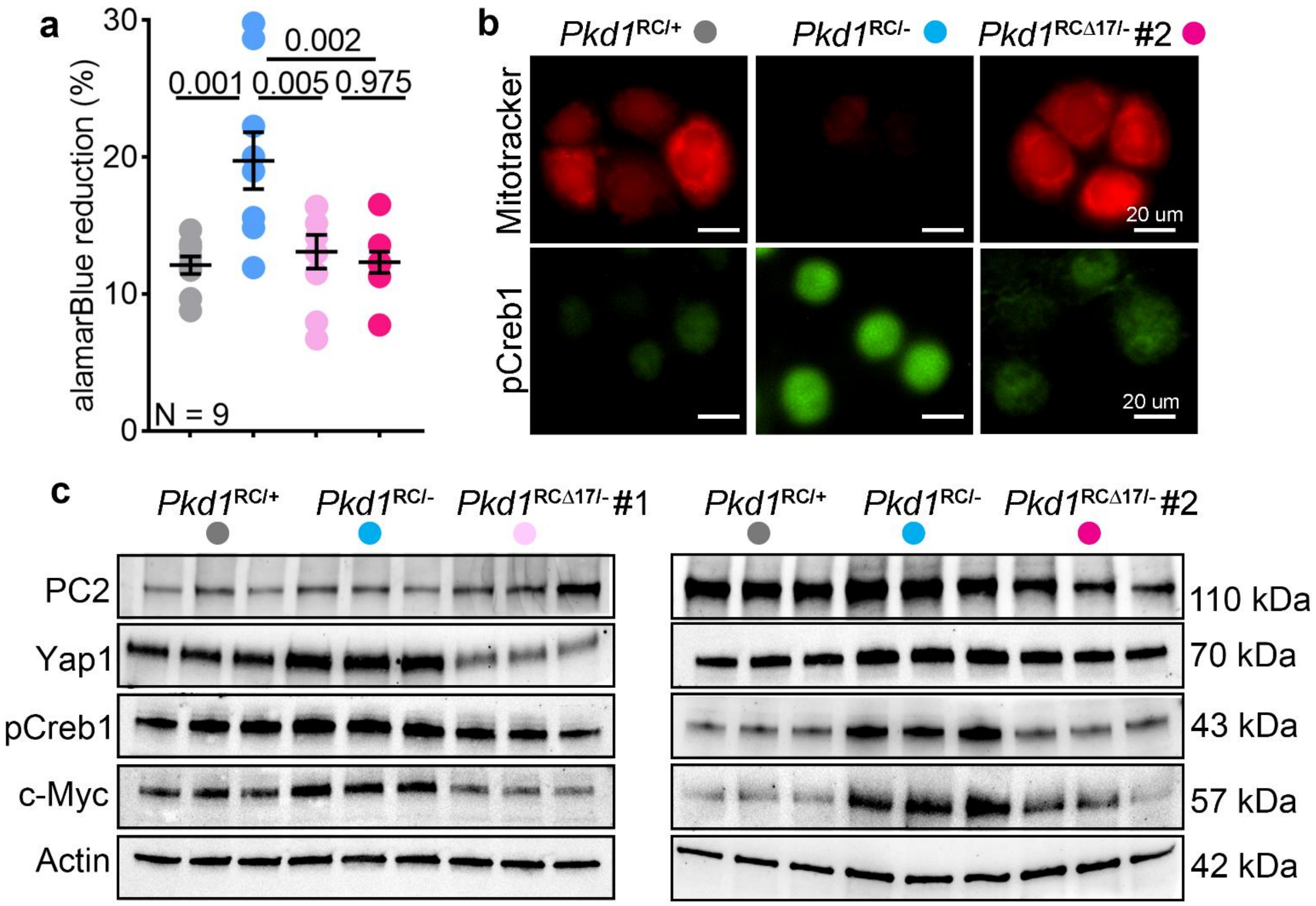

Supplementary Figure 6. Phenotypic characterization of CRISPR-edited *Pkd1$^{RC\Delta17}$* cell lines. a. Alamarblue assay showing reduced proliferation of *Pkd1* clones which lack miR-17 motif compared to *Pkd1*$^{RC/-}$ parent cell line at 12 hours. *n* = 9 biologically independent experiments. **b.** Mitotracker images and IF staining for pCreb1 showing restored mitochondrial membrane potential (red) and reduced pCreb1 (green) expression in *Pkd1*$^{RC\Delta17/-}$ clone #2 compared to the parental cell line. *n* = 3 biologically independent experiments. **c.** Western blot characterization of both *Pkd1*$^{RC\Delta17/-}$ cell lines showing reduced expression of cyst promoting genes Yap1, pCreb1, and c-Myc compared to parental *Pkd1*$^{RC/-}$ cells. As a pertinent control, PC2 expression remained unchanged. Actin serves as the loading control. *n* = 3 biologically independent experiments. Error bars indicate SEM. Statistical analysis: one-way ANOVA, Tukey's multiple comparisons test (a).

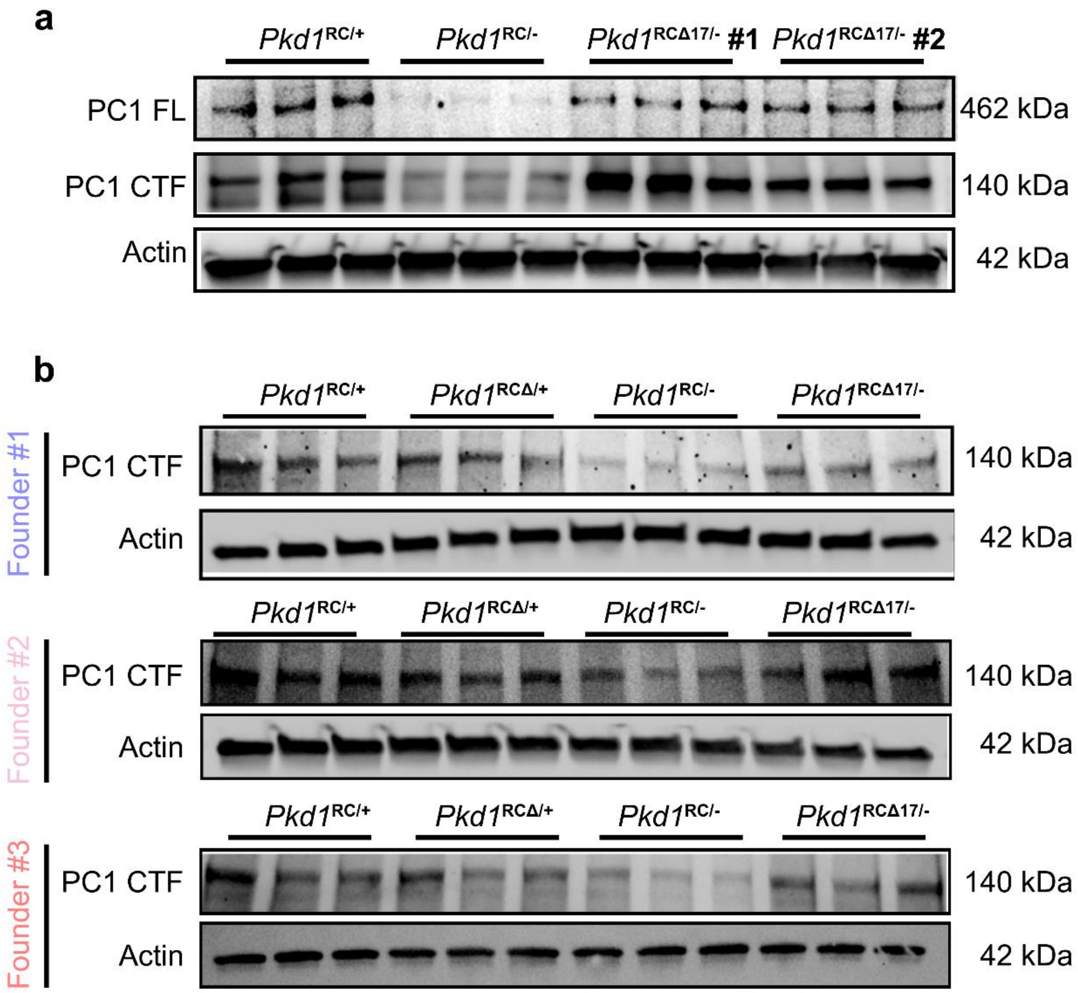

Supplemental Figure 7. PC1 derepression in CRISPR-edited *Pkd1*$^{RC\Delta17/-}$ cell lines and mouse kidneys. We used two independent PC1 antibodies (7E12 from Santa Cruz and E8-8C3C10 from U Maryland) to examine PC1 expression in *Pkd1*$^{RC\Delta17/-}$ cell lines and kidneys. The 7E12 antibody detects the full-length 462 kDa PC1, whereas the E8-8C3C10 antibody detects the 140 kDa c-terminal fragment (CTF) of the PC1 protein. **a.** Immunoblot showing full-length PC1 and PC1-CTF expression in the *Pkd1*$^{RC\Delta17/-}$ clones #1 and #2 compared to the control *Pkd1*$^{RC/+}$ and *Pkd1*$^{RC/-}$. n = 3 biologically independent experiments. **b.** Immunoblots using the E8 antibody showing PC1 expression in the kidneys of mice with the indicated genotypes. Analysis of progeny from all three founders (#1, #2, and #3) is shown. PC1 western blots using the 7E12 antibody are shown in Figure 2. Both antibodies exhibit consistent results in cells and kidney tissue. Actin is used as the loading control. n = 3 biologically independent kidney samples for each genotype.

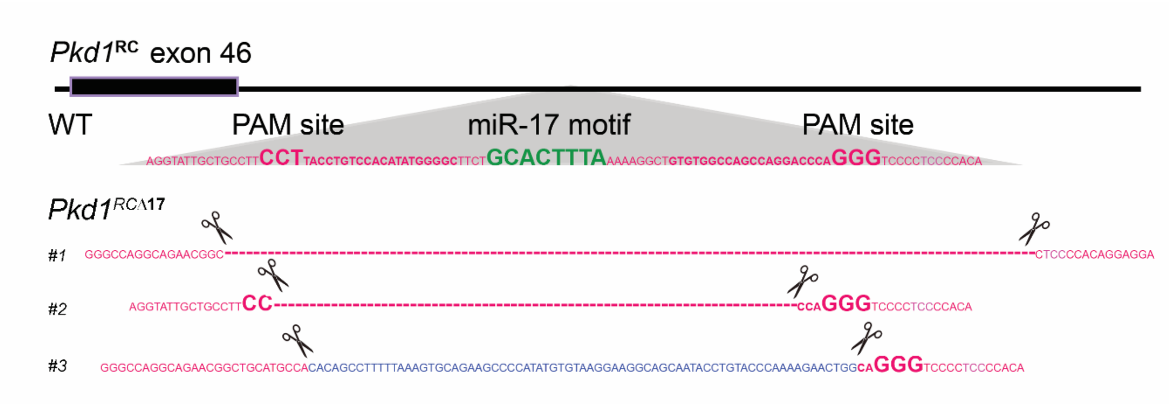

Supplementary Figure 8. **Characterization of CRISPR-edited *Pkd1*$^{RC/RC}$ mice**. Graphical illustration of Sanger sequencing from tail DNA from the three CRISPR-edited founders. Founders #1 and #2 harbor 108 bp and 53 base pair deletions, respectively, including the miR-17 motif. Founder #3 also lacked the miR-17 motif but acquired a 72 base pair insertion (blue), resulting in a net loss of 18 base pairs in the 3'-UTR sequence.

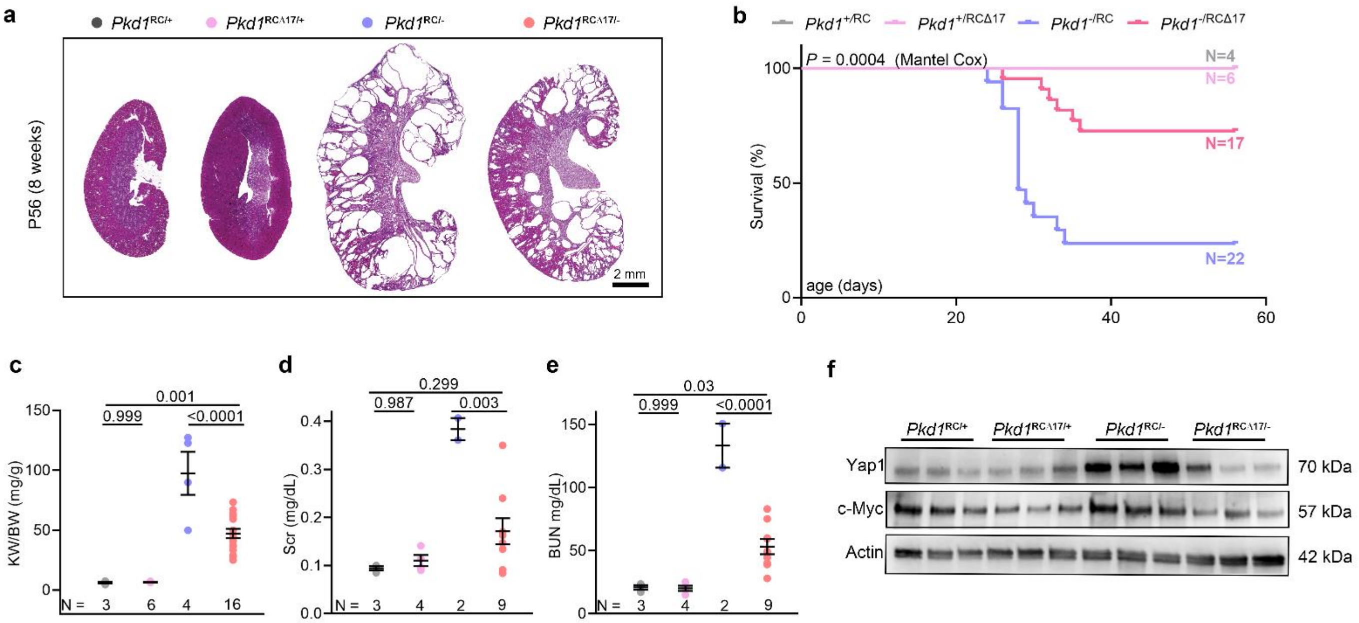

Supplementary Figure 9: Monoallelic *Pkd1* derepression suppresses disease progression. A cohort of progeny derived from founder #2 was prospectively monitored till eight weeks of age. **a.** H&E-stained kidney sections of mice of the indicated genotypes that survived till eight weeks of age are shown. *Pkd1*$^{RC/+}$ (n = 3), *Pkd1*$^{RC\Delta17/+}$ (n = 3), *Pkd1*$^{RC/-}$ (n = 4), and *Pkd1*$^{RC\Delta17/-}$ (n = 16). **b.** Kaplan-Meir survival curves of mice with the indicated genotypes are shown. **c-e**. KW/BW, serum creatinine (Scr), and BUN levels of the surviving 8-week-old mice with indicated genotypes are shown. **f**. Immunoblots showing Yap1 and c-Myc expression in kidneys of 18-day-old mice with the indicated genotypes (n = 3 for all genotypes). Actin serves as the loading control. Error bars indicate SEM. Statistical analysis: one-way ANOVA, Tukey's multiple comparisons test (c-e); Log-rank Mantel-Cox (b).

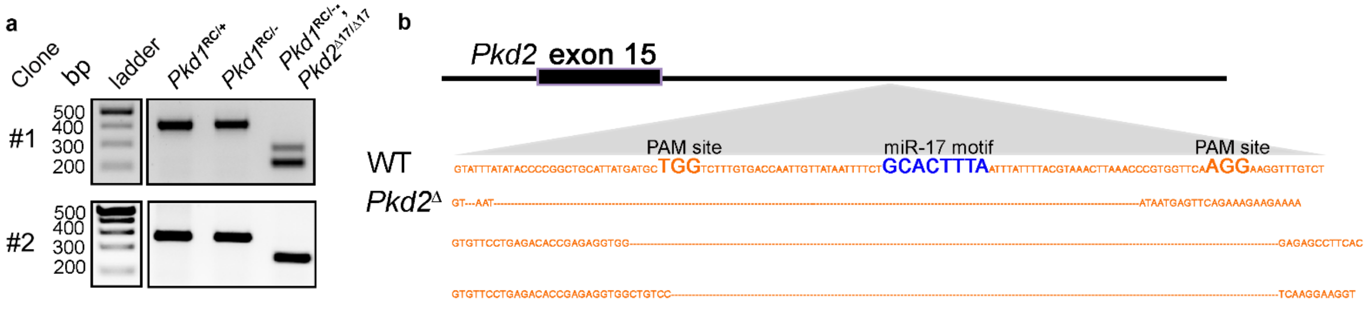

Supplementary Figure 10: Characterization of *Pkd1*$^{RC/-}$ cell lines lacking miR-17 motif from *Pkd2* 3'-UTR. **a.** PCR products obtained after amplifying the DNA (encoding the *Pkd2* 3'UTR segment) from parental and CRISPR-edited cell lines. The lower bands indicate the Δ17 genotype. **b.** Graphical illustration of Sanger sequencing results from the Δ17 bands of each clone confirm deletion of the miR-17 motif from both *Pkd2* alleles.

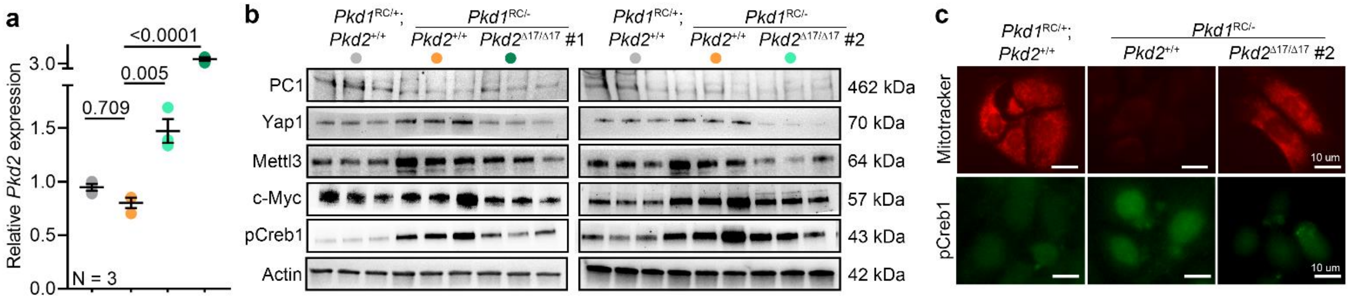

Supplemental Figure 11. Phenotypic characterization of CRISPR-edited *Pkd1$^{RC/-}$; Pkd2*$^{\Delta17/\Delta17}$ cell lines. **a.** qRT-PCR showing *Pkd2* derepression in both *Pkd1*$^{RC/-}$*; Pkd2*$^{\Delta17/\Delta17}$ clones #1 and #2 compared to the parental *Pkd1*$^{RC/-}$ cell line. *n* = 3 biologically independent samples from the indicated cell lines. **b.** Western blot characterization shows a reduced expression of cyst-promoting genes Yap1, Mettl3, c-Myc, and pCreb1 in both *Pkd1*$^{RC/-}$*; Pkd2*$^{\Delta17/\Delta17}$ clones compared to the parent *Pkd1*$^{RC/-}$ cell line. Pertinently, PC1 expression remained unchanged. Actin serves as a loading control. *n* = 3 biologically independent samples from the indicated cell lines. **c**. Mitotracker images and IF staining for pCreb1 showing restored mitochondrial membrane potential (red) and reduced pCreb1 (green) expression, respectively, in the *Pkd1*$^{RC/-}$*; Pkd2*$^{\Delta17/\Delta17}$ clone #2 compared to the parental cell line. *n* = 3 biologically independent samples from the indicated cell lines. Error bars indicate SEM. Statistical analysis: one-way ANOVA, Tukeys' multiple comparisons test (a).

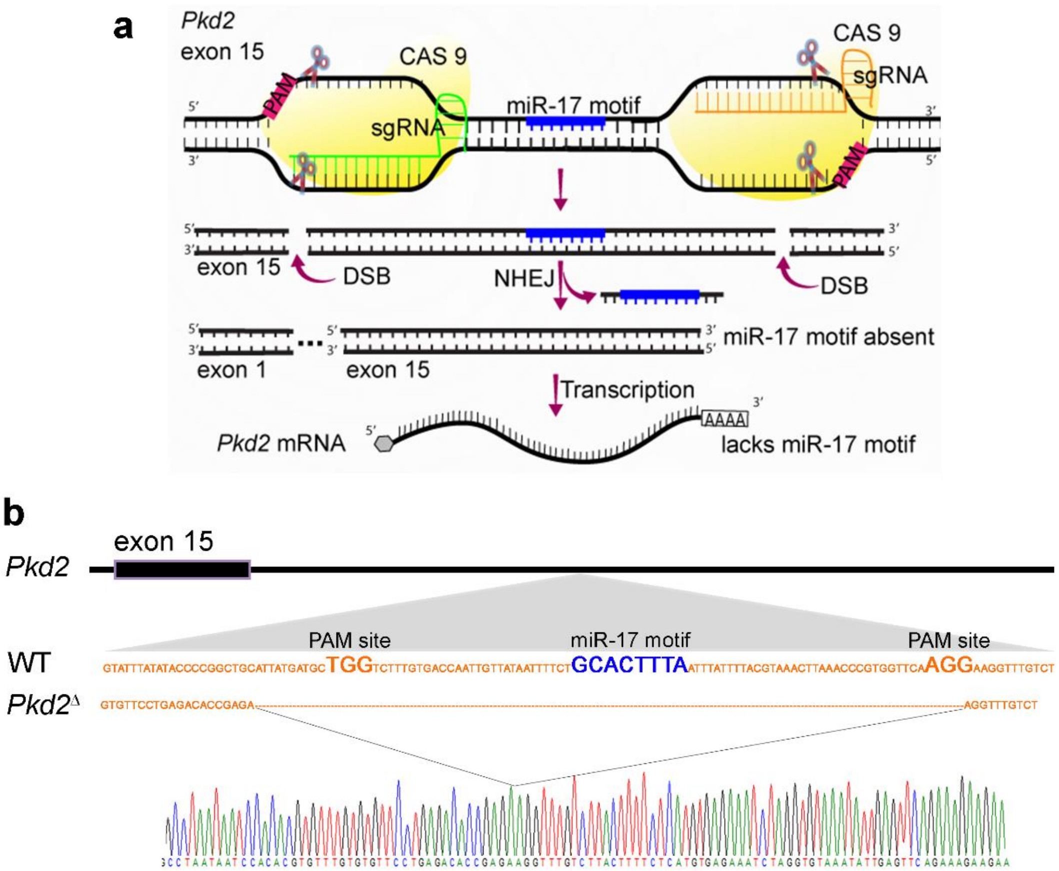

Supplementary Figure 12. **CRISPR-editing and characterization of** *Pkd2*$^{\Delta 17}$ **mice that lack miR-17 motif in *Pkd2* 3'-UTR. a.** Graphic illustrates the CRISPR-editing strategy used to remove the miR-17 motif from exon 15 of *Pkd2*. **b**. Graphical illustration and Sanger sequencing results of PCR product from the tail DNA of founder mouse showing 139 base pair deletion from the *Pkd2* 3'-UTR, including the miR-17 motif.

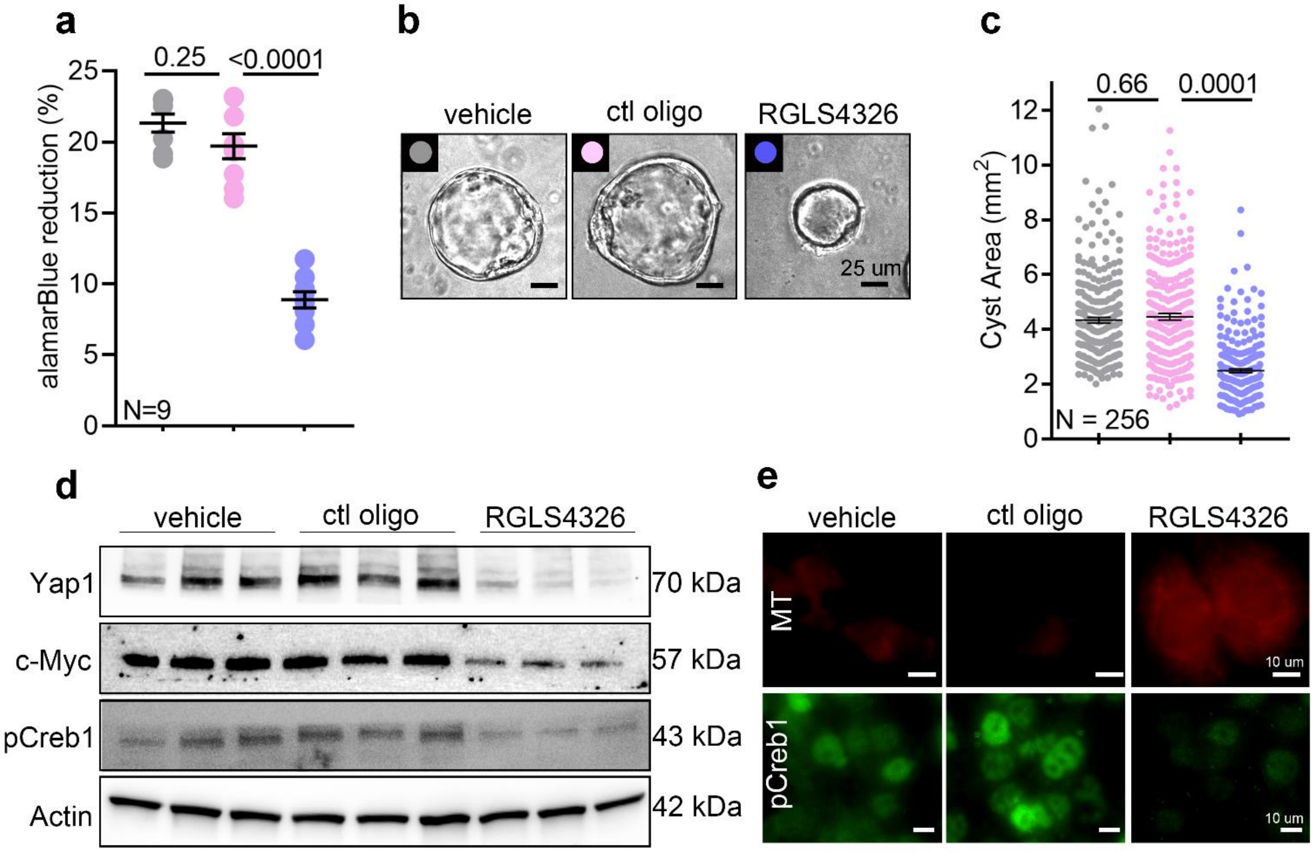

Supplementary Figure 13: Phenotypic characterization of RGLS4326 treatment on *Pkd1*$^{RC/-}$ cells. *Pkd1*$^{RC/-}$ cells were transfected with 100 uM RGLS4326, 100 uM control oligonucleotide, or vehicle control. Seventy-two hours later, cells were equivalently seeded in 96 well plate for 12 hours for the alamarBlue assay or placed in matrigel for seven days for a 3D cyst assay. **a.** Reduced proliferation of *Pkd1*$^{RC/-}$ cells treated with RGLS4326 compared to cells treated with control oligonucleotide or vehicle control. *n* = 9 biologically independent experiments for each treatment group. **b-c.** Representative images and quantification show a reduction in cyst size of RGLS4326-treated cells compared to vehicle or control oligonucleotide-treated *Pkd1*$^{RC/-}$ cells. No significant change was observed in cyst size between vehicle control and control oligonucleotide-treated groups. **d.** Immunoblots showing Yap1, c-Myc, and pCreb1 expression in *Pkd1*$^{RC/-}$ transfected with a vehicle, control oligonucleotide or RGLS4326. Actin is the loading control. *n* = 3 biologically independent experiments for each treatment group. **e.** Mitotracker labeling and anti-pCreb1 immunostaining of *Pkd1*$^{RC/-}$ cells transfected with a vehicle, control oligonucleotide, or RGLS4326. *n* = 3 biologically independent experiments for each treatment group. Error bars indicate SEM. Statistical analysis: one-way ANOVA, Tukeys' multiple comparisons test (a,c).

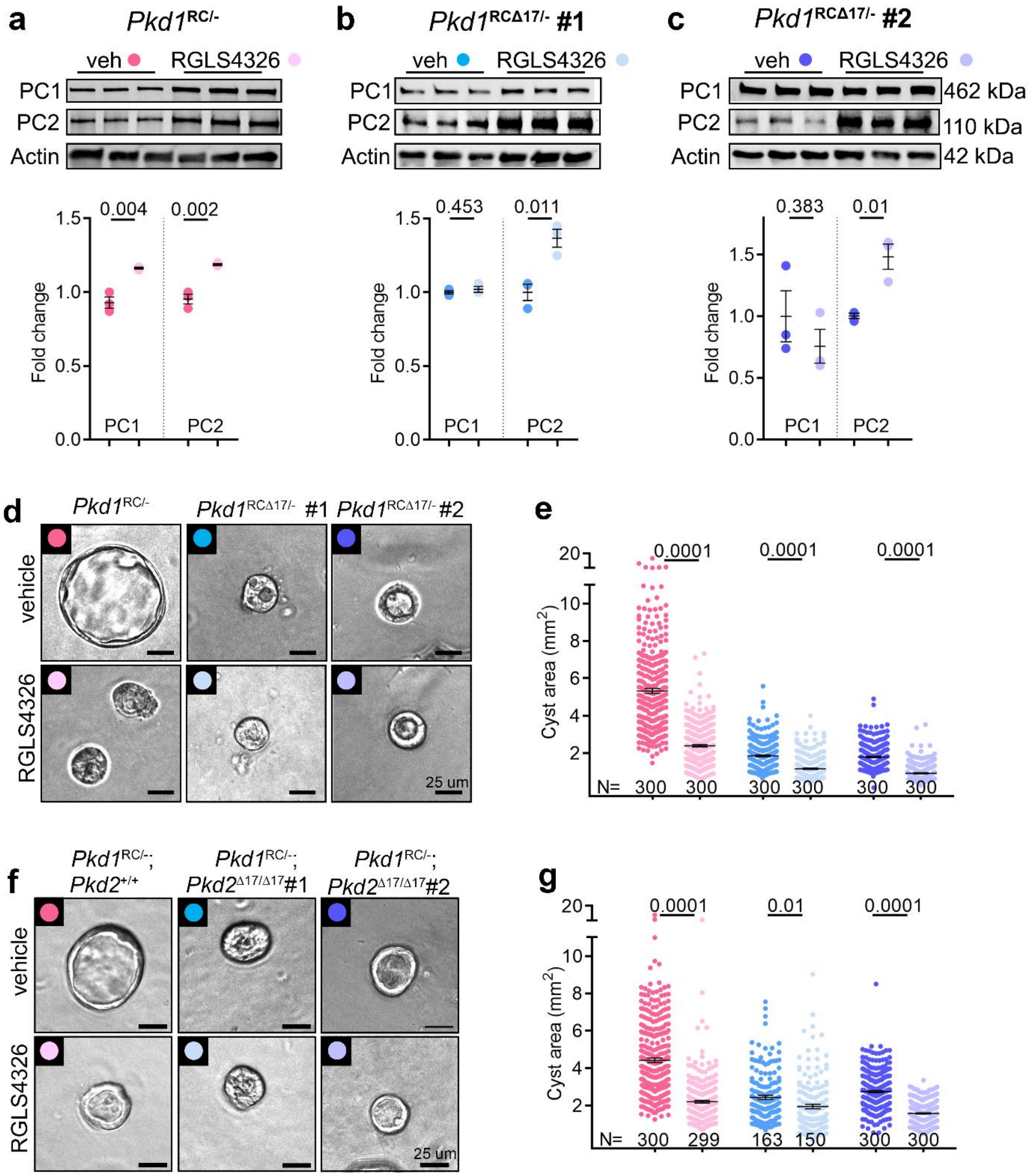

Supplementary Figure 14: Characterization of RGLS4326 treatment on CRISPR-edited *Pkd1*$^{RC\Delta17/-}$ or *Pkd1*$^{RC/-}$*; Pkd2*$^{\Delta17/\Delta17}$ cell lines. a-c. *Pkd1*$^{RC/-}$ (a), *Pkd1*$^{RC\Delta17/-}$ clone#1 (b), and *Pkd1*$^{RC\Delta17/-}$ clone#2 (c) cells were transfected with 100 uM RGLS4326 or vehicle control. Seventy-two hours later, protein from these cells was analyzed by western blot to assess PC1 and PC2 expression. (a) Expression of PC1 and PC2 was increased in the *Pkd1*$^{RC/-}$ cells upon RGLS4326 treatment. (b and c) No additional PC1 upregulation was observed after RGLS4326 treatment in the *Pkd1*$^{RC\Delta17/-}$ clones #1 or #2, indicating that this oligo medicates PC1 derepression via the miR-17 motif in the *Pkd1* 3'-UTR. As expected, PC2 expression was increased in

the *Pkd1*$^{RC\Delta17/-}$ clones #1 and #2 with RGLS4326 treatment. Actin serves as the loading control. Quantification of the western blots is shown in the graphs below (*n* = 3 biologically independent samples from each of the indicated cell lines and treatment groups). **d-e.** Representative images and quantification showing cyst size of vehicle or 100 uM RGLS4326-treated *Pkd1*$^{RC/-}$, *Pkd1*$^{RC\Delta17/-}$ (clone#1), or *Pkd1*$^{RC\Delta17/-}$ (clone#2). **f-g**. Representative images and quantification showing cyst size of vehicle or 100 uM RGLS4326-treated *Pkd1*$^{RC/-}$, *Pkd1*$^{RC/-}$; *Pkd2*$^{\Delta17/\Delta17}$ (clone#1) or *Pkd1*$^{RC/-}$; *Pkd2*$^{\Delta17/\Delta17}$ (clone#2). Error bars indicate SEM. Statistical analysis: Two-tailed Student's T-test (a-c); one-way ANOVA, Tukeys' multiple comparisons test (e and g).

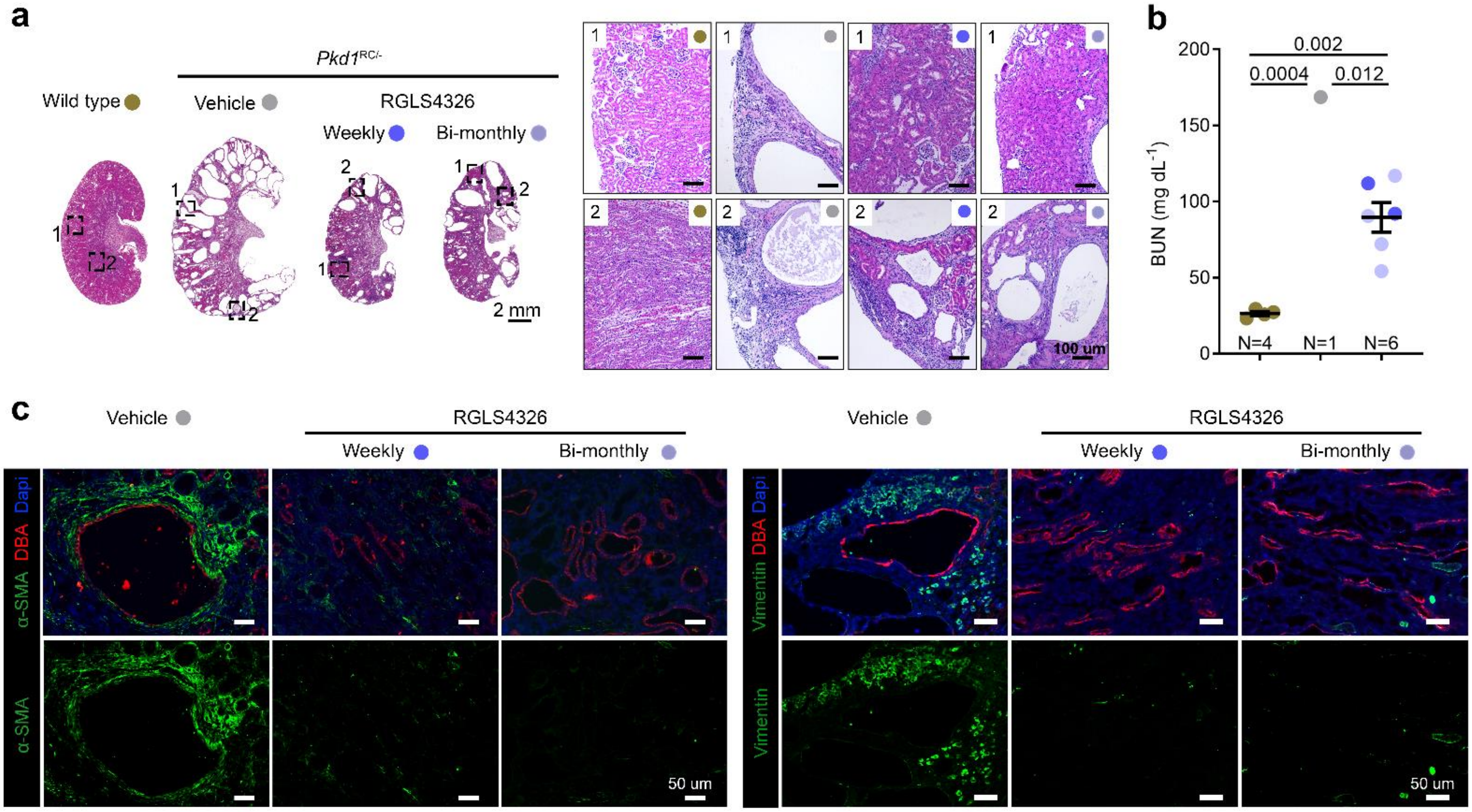

Supplementary Figure 15. Characterization of long-term RGLS4326 treatment in *Pkd1*$^{RC/-}$ mice **a.** Low and high magnification images of H&E-stained kidneys from P125-day-old *Pkd1*$^{RC/-}$ mice treated with vehicle or RGLS4326 are shown. The high magnification images are from the regions marked by black insets on the low magnification images. Substantially preserved histology was observed in kidneys of *Pkd1*$^{RC/-}$ mice treated with RGLS4326 compared to vehicle. **b.** BUN levels of *Pkd1*$^{RC/-}$ mice treated with vehicle (grey, n = 1) or RGLS4326 (shades of blue, n = 6). BUN from age-matched, non-cystic controls (brown, n = 4) is shown as a reference of the normal values. The comparison is limited because the blood from only one of the two surviving vehicle-treated *Pkd1*$^{RC/-}$ mice was available for renal function measurement. BUN levels are not available for the control oligo-treated *Pkd1*$^{RC/-}$ mice because none of them survived till the end of the study at 125 days. **c.** IF staining showing reduced expression of fibrosis markers, α-SMA and Vimentin in kidneys from *Pkd1*$^{RC/-}$ mice treated with RGLS4326 compared to vehicle (*n = 2* for vehicle, *n* = 3 for each treatment group). Statistical analysis: ANOVA, Tukeys' multiple comparisons test (b).

Donor	Gene	cDNA	Amino Acid	Exon	Type
1	*PKD1*	c.6657_6671dupGCGGCTGGCGCTGCC	p.R2220_P2224dup	15	Insertion
2	*PKD1*	c.8755G>A	p.G2919R	23	Missense
	PKD2	c.2419C>T	p.R807*	13	Truncating
3	*PKD1*	c.11775dupG	p.R3926Afs*34	43	Truncating
4	*PKD1*	c.7921C>T	p.Q2641*	21	Truncating

Supplementary Figure 16. Mutation analysis on kidney cyst cells of ADPKD donors. Genomic DNA from ADPKD donor cyst cells was used to perform *PKD1* and *PKD2* mutation analysis. DNA sequencing and mutation analysis was performed by Ambry Genetics. The details of identified mutations in the four donor cell lines are shown.

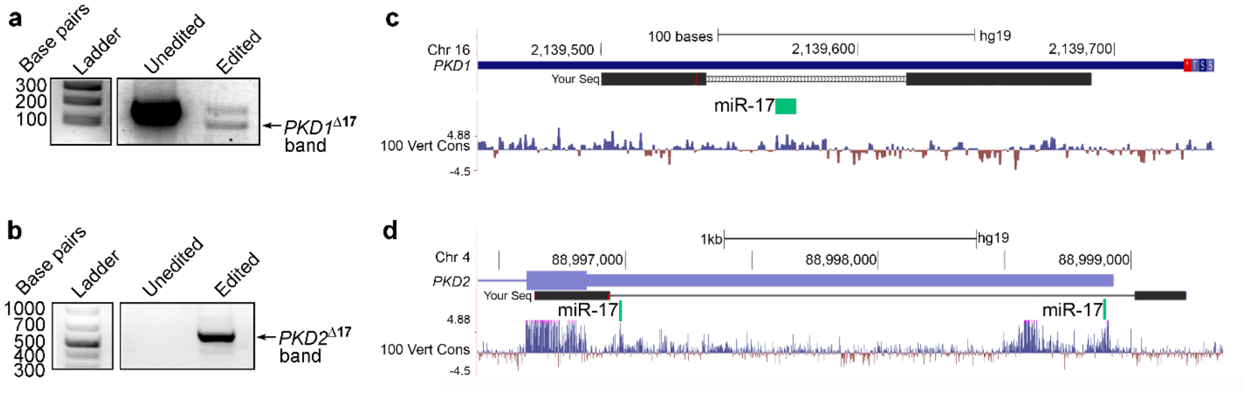

Supplementary Figure 17: Genotyping of CRISPR-edited primary human ADPKD cultures. a-b. PCR products obtained after amplifying the DNA encoding the *PKD1* (**a**) or *PKD2* (**b**) 3'UTR segment from unedited parental and CRISPR-edited human ADPKD cultures. The arrows indicate the PCR bands resulting from miR17 motif deletion in *PKD1* (**a**) and *PKD2* (**b**) genes. **c-d.** Sanger sequencing of the PCR product (black rectangles) aligned with the human genome (purple rectangles). The deleted region contains the miR-17 binding site (green rectangles).

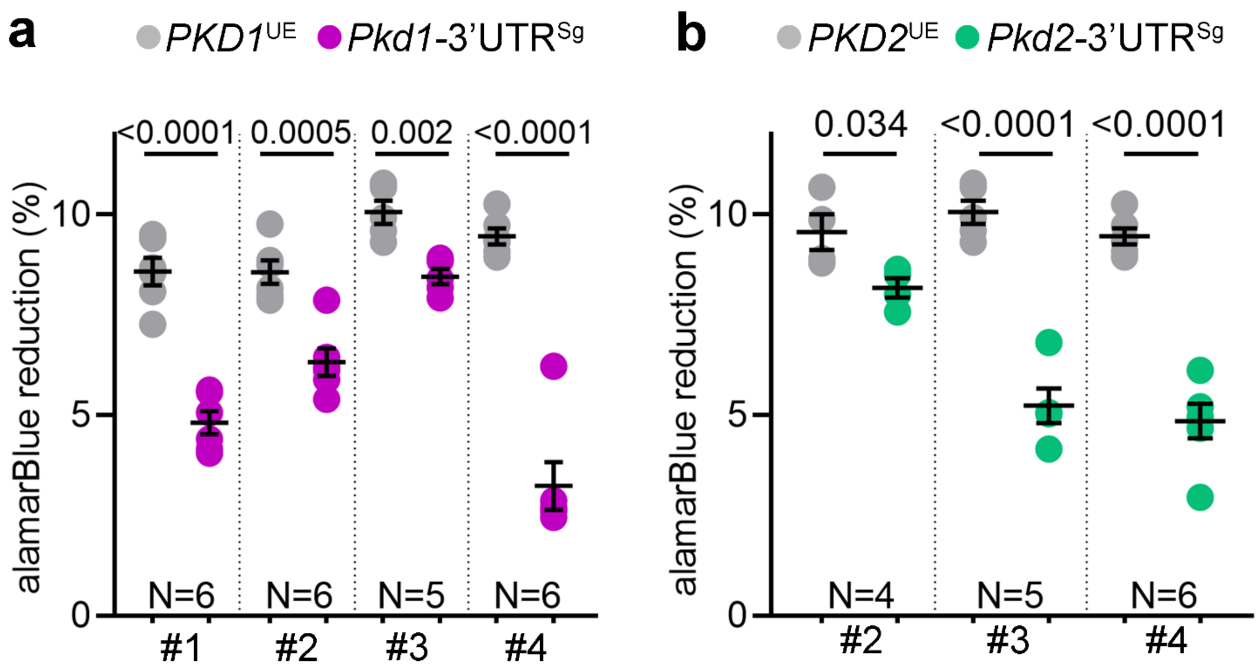

Supplementary Figure 18: Reduced proliferation in *PKD1*$^{\Delta 17}$ and *PKD2*$^{\Delta 17}$ edited ADPKD cultures. **a-b.** alamarBlue-assessed proliferation of ADPKD donor cultures that were CRISPR-edited to remove the miR-17 motif in either the *PKD1* (pink) or *PKD2* (Green) gene compared to their respective unedited (UE) parental controls (grey). Error bars indicate SEM. *n*'s indicate biologically independent experiments for each cell line and treatment. Statistical test: Two-tailed Students *t*-test for each cell line separately.

**Cell line**	***Pkd1***$^{RC/-}$		***Pkd1***$^{RC\Delta17/-}$ **#1**		***Pkd1***$^{RC/-}$		***Pkd1***$^{RC\Delta17/-}$ **#1**		***Pkd1***$^{RC/-}$		***Pkd1***$^{RC\Delta17/-}$ **#1**	
**Stressor**	Ctl	cAMP	Ctl	cAMP	Ctl	Gluc	Ctl	Gluc	Ctl	SAM	Ctl	SAM
**Replicate**												
1	40.19	46.67	30.04	28.88	38.07	49.99	27.39	27.35	38.58	47.47	26.87	29.91
2	39.75	45.56	25.8	25.63	37.34	49.78	31.51	27.74	39.09	46.37	25.20	26.70
3	40.32	41.16	26.19	26.97	38.19	47.97	28.27	27.03	39.29	42.01	27.25	28.02
4	37.42	42.82	27.03	27.97	37.78	44.98	27.62	26.04	37.77	43.66	28.09	29.01
5	38.77	40.43	27.26	28.99	34.85	43.70	27.25	28.45	37.33	41.29	28.32	30.03
6	36.75	42.37	27.79	28.19	38.74	43.92	25.34	28.74	40.58	43.21	28.85	29.24
7	37.98	44.11	25.84	26.91	40.26	46.17	25.54	29.29	39.85	44.93	26.91	27.97
8	39.15	41.98	27.92	22.64	38.77	48.13	28.19	29.96	39.51	42.83	28.98	23.72
***P* value**												
Ctl vs Stressor	0.0002				<0.0001				<0.0001			
Ctl vs Stressor			.9952				.7041				.9296	
Ctl vs Ctl	<0.0001				<0.0001				<0.0001			

Supplementary Table 1. alamarBlue assay on *Pkd1*$^{RC/-}$ and *Pkd1*$^{RC\Delta17/-}$ #1 cells. Raw values of percentage reduction are shown for each biological replicate. The results of one-way ANOVA followed by post-hoc Tukey's multiple comparisons test are listed.

**Cell line**	***Pkd1***$^{RC/-}$		***Pkd1***$^{RC/-}$ ***Pkd2***$^{\Delta17/\Delta17}$ **#1**		***Pkd1***$^{RC/-}$		***Pkd1***$^{RC/-}$ ***Pkd2***$^{\Delta17/\Delta17}$ **#1**		***Pkd1***$^{RC/-}$		***Pkd1***$^{RC/-}$ ***Pkd2***$^{\Delta17/\Delta17}$ **#1**	
**Stressor**	Ctl	cAMP	Ctl	cAMP	Ctl	Gluc	Ctl	Gluc	Ctl	SAM	Ctl	SAM
**Replicate**												
1	32.32	36.36	18.7	17.73	27.94	34.09	23.44	21.66	38.11	50.04	29.66	31.93
2	32.14	36.15	17.51	18.6	28.8	34.05	21.61	21.57	43.64	51.71	29.57	30.41
3	30.55	36.5	17.79	19	29.57	34.49	22.48	19.47	42.4	50.53	31.2	30.42
4	30.43	37.4	19.85	17.84	29.49	34.34	20.42	20.87	43.66	51.62	30.76	32.56
5	31.35	37.14	19.25	19.67	28.05	35.09	18.43	21.22	43.51	48.1	30.62	32
6	32.44	38.96	19.17	19.8	27.4	35.16	19.93	21.22	43.83	53.31	30.21	32.06
7	32.28	36.35	19.06	21.36	28.29	35.84	20.94	18.87	42.98	49.38	30.31	30.7
8	32.55	38.71	18.34	17.82	27.86	36.1	19.45	20.13	43.6	48.67	33.71	30.95
***P* value**												
Ctl vs Stressor	<0.0001				<0.0001				<0.0001			
Ctl vs Stressor			.9519				.9811				.8421	
Ctl vs Ctl	<0.0001				<0.0001				<0.0001			

Supplementary Table 2. alamarBlue assay on *Pkd1*$^{RC/-}$ and *Pkd1*$^{RC/-}$;*Pkd2*$^{\Delta17/\Delta17}$ #1 cells. Raw values of percentage reduction are shown for each biological replicate. The results of one-way Anova followed by post-hoc Tukey's multiple comparisons test are listed.

	sgRNA sequence (5'-3')		Genotyping Primers (5'-3')	
	**Upstream**	**Downstream**	**Forward Primer**	**Reverse Primer**
Pkd1	GCCCATATGTGGACAGGTA	GTGTGGCCAGCCAGGACCCA	CTAGGGGTCTTGGCCATTCC	CTGAACCTGAGGACTTGGGG
Pkd2	CGAACTTATCATTGTCGTAC	AAACTTAAACCCGTGGTTCA	GTTCCTGAGACACCGAGAGG	GGCAGCAAATCAGCGTACTT
PKD1	ACGTAGGTTCCCCAGAGAGCA	TGTGTGGCCAACCAGGACCC	TGAGGACTCGGGGAAATAAA	CGTGGAGTCGGAGTGGAC
PKD2	TTATATGCCCTGACCACCAT	AATGTTTTCGGACATAGAGA	GGTCGTGACAGTGAAATCCA	(WT) CAGAAGATTAGCAATCGGTCAC (Deletion Specific) TGGCAGCCTATGCTGTTATG

Supplementary Table 3. sgRNA Sequences and Genotyping Primer Sequences

	Forward (5'-3')	Reverse (5'-3')
Pkd1	CTAGACCTGTCCCACAACCTA	GCAAACACGCCTTCTTCTAATGT
Pkd2	GCGTGGTACCCTCTTGGCAGTT	CACGACAATCACAACATCC
Pkd1 (+ allele)	CATATGGGGCTTCTGCACTT	GAGGCTGGGTACTCACTTGG
Pkd1 (∆ allele)	ATTGCTGCCTTCCTTACCCC	CTGGGTACTCACTTGGTCCA

Supplementary Table 4. Q-PCR primer sequences
