# Supplementary material for: PKD1 and PKD2 mRNA cis-inhibition drives polycystic kidney disease progression
Source: Nat Commun. 2022 Aug 15;13:4765. doi: 10.1038/s41467-022-32543-2 (PMC9376183; doi:10.1038/s41467-022-32543-2)
Supplement: Supplementary file 2 — Reporting Summary [file 41467_2022_32543_MOESM2_ESM.pdf]

nature portfolio

Corresponding author(s): Vishal Patel

Last updated by author(s): Jun 16, 2022

Reporting Summary

Nature Portfolio wishes to improve the reproducibility of the work that we publish. This form provides structure for consistency and transparency in reporting. For further information on Nature Portfolio policies, see our Editorial Policies and the Editorial Policy Checklist.

Statistics

For all statistical analyses, confirm that the following items are present in the figure legend, table legend, main text, or Methods section.

n/a	Confirmed	
☐	☒	The exact sample size (*n*) for each experimental group/condition, given as a discrete number and unit of measurement
☐	☒	A statement on whether measurements were taken from distinct samples or whether the same sample was measured repeatedly
☐	☒	The statistical test(s) used AND whether they are one- or two-sided<br>*Only common tests should be described solely by name; describe more complex techniques in the Methods section.*
☐	☒	A description of all covariates tested
☐	☒	A description of any assumptions or corrections, such as tests of normality and adjustment for multiple comparisons
☐	☒	A full description of the statistical parameters including central tendency (e.g. means) or other basic estimates (e.g. regression coefficient) AND variation (e.g. standard deviation) or associated estimates of uncertainty (e.g. confidence intervals)
☐	☒	For null hypothesis testing, the test statistic (e.g. *F*, *t*, *r*) with confidence intervals, effect sizes, degrees of freedom and *P* value noted<br>*Give P values as exact values whenever suitable.*
☒	☐	For Bayesian analysis, information on the choice of priors and Markov chain Monte Carlo settings
☒	☐	For hierarchical and complex designs, identification of the appropriate level for tests and full reporting of outcomes
☐	☒	Estimates of effect sizes (e.g. Cohen's *d*, Pearson's *r*), indicating how they were calculated

Our web collection on statistics for biologists contains articles on many of the points above.

Software and code

Policy information about availability of computer code

Data collection	Sequencing quality control was performed with FastQC v0.11.8. RNA-seq reads were trimmed, and low-quality reads were removed using Trimgalore v0.6.3_dev (https://www.bioinformatics.babraham.ac.uk/projects/trim_galore/) with the "paired" parameter and length of 150 bps. Trimmed fastq sequences were aligned to the mouse reference genome GRCm38 using STAR aligner v2.5.3a with the produced barn files sorted by coordinate by using the option "--outSAMtype BAM SortedByCoordinate." Raw read gene counts were obtained using STAR aligner with options "-quantMode GeneCounts" and "--sjdbGTFfile" with gene models in GTF format obtained from mouse EnsEMBL release 94. Alignment quality control and read mapping statistics were obtained from Picard tools v2.20.3 using the function "CollectMu ltipleMetrics" (http://broadinstitute.github.io/picard/).
Data analysis	Raw gene counts were used for quality control and differential expression analysis. Raw counts were normalized to the total number of reads by calculating log2CPM (counts per million), and lowly expressed genes (average Log2CPM < -3) were eliminated before differential gene expression analysis. TPM (transcript per million) quantification was done using RSEM vl.3.1, and differential gene expression analysis was performed using the limma-trend (version 3.40.6) in R.

For manuscripts utilizing custom algorithms or software that are central to the research but not yet described in published literature, software must be made available to editors and reviewers. We strongly encourage code deposition in a community repository (e.g. GitHub). See the Nature Portfolio guidelines for submitting code & software for further information.

Data

Policy information about availability of data

All manuscripts must include a data availability statement. This statement should provide the following information, where applicable:

- Accession codes, unique identifiers, or web links for publicly available datasets
- A description of any restrictions on data availability
- For clinical datasets or third party data, please ensure that the statement adheres to our policy

RNA-Seq Acession code: GSE196237. No restrictions

Human research participants

Policy information about studies involving human research participants and Sex and Gender in Research.

Reporting on sex and gender	NA
Population characteristics	NA
Recruitment	NA
Ethics oversight	NA

Note that full information on the approval of the study protocol must also be provided in the manuscript.

Field-specific reporting

Please select the one below that is the best fit for your research. If you are not sure, read the appropriate sections before making your selection.

- [x] Life sciences
- [ ] Behavioural & social sciences
- [ ] Ecological, evolutionary & environmental sciences

For a reference copy of the document with all sections, see nature.com/documents/nr-reporting-summary-flat.pdf

Life sciences study design

All studies must disclose on these points even when the disclosure is negative.

Sample size	The sample size was determined based on our previous experience working with these animal models
Data exclusions	No data were excluded
Replication	Each experiment had at least three biological replicates and were repeated at least three times (technical replicates).
Randomization	Animals were randomly assigned to treatment arms.
Blinding	Investigators were not blinded to the treatment or the genotypes of animals.

Reporting for specific materials, systems and methods

We require information from authors about some types of materials, experimental systems and methods used in many studies. Here, indicate whether each material, system or method listed is relevant to your study. If you are not sure if a list item applies to your research, read the appropriate section before selecting a response.

Materials & experimental systems

n/a	Involved in the study	
☐	☒	Antibodies
☐	☒	Eukaryotic cell lines
☒	☐	Palaeontology and archaeology
☐	☒	Animals and other organisms
☒	☐	Clinical data
☒	☐	Dual use research of concern

Methods

n/a	Involved in the study	
☒	☐	ChIP-seq
☒	☐	Flow cytometry
☒	☐	MRI-based neuroimaging

Antibodies

Antibodies used	DBA (Vector Labs, catalog# B-1035), THP (Biomedical Technologies, catalog# BT-590), LTA (Vector Labs, catalog# B-1325), MRCl (Abeam, catalog# ab64693), pCREBl (Cell Signaling, catalog# 9198); pHH3 (Sigma, catalog# H0412), PCl (7E12 Santa Cruz, catalog# sc-130554); PC2 (gift from the Baltimore PKD Core); pCREB (Cell Signaling, catalog# 9198); c-Myc (Abeam, catalog# ab185656), YAPl {Cell Signaling, catalog# 4912); Mettl3 (lnvitrogen, catalog# MAS-27527) ;PC1 E8-8C3C10 (Baltimore PKD core center)
Validation	These are validated antibodies; We validated the PC1 antibodies in our Pkd1-null cell lines.

Eukaryotic cell lines

Policy information about cell lines and Sex and Gender in Research

Cell line source(s)	Primary human ADPKD cyst cells were obtained from PKD Research Biomarker and Biomaterial Core at the University of Kansas Medical Center (KUMC). The use of surgically discarded kidney tissues complied with federal regulations and was approved by the Institutional Review Board at the University of Kansas Medical Center.
Authentication	Not authenticated
Mycoplasma contamination	The cells were not tested for mycoplasma
Commonly misidentified lines (See ICLAC register)	NA

Animals and other research organisms

Policy information about studies involving animals; ARRIVE guidelines recommended for reporting animal research, and Sex and Gender in Research

Laboratory animals	All mice were maintained in C57BL/6J background. At pre-specified time points, mice were anesthetized using an approved protocol, and blood was obtained via cardiac puncture. All studies used equal males and females. The UT Southwestern Institutional Animal Care and Use Committee approved all experiments involving animals.
Wild animals	NA
Reporting on sex	Findings apply to both sexes. Sex was considered in the design of the studies. The sex breakdown for the data is provided in the source data files.
Field-collected samples	NA
Ethics oversight	All experiments involving animals were approved by the UT Southwestern Institutional Animal Care and Use Committee.

Note that full information on the approval of the study protocol must also be provided in the manuscript.
